# Specific immune response to *M. tuberculosis* and ability to *in vitro* control mycobacterial replication are not impaired in subjects with immune-mediated inflammatory disease and tuberculosis infection

**DOI:** 10.3389/fimmu.2024.1484143

**Published:** 2025-01-13

**Authors:** Chiara Farroni, Anna Maria Gerarda Altera, Andrea Salmi, Valentina Vanini, Gilda Cuzzi, Cecilia S. Lindestam Arlehamn, Alessandro Sette, Giovanni Delogu, Ivana Palucci, Settimia Sbarra, Alessandra Aiello, Andrea Picchianti-Diamanti, Gina Gualano, Fabrizio Palmieri, Delia Goletti, Elisa Petruccioli

**Affiliations:** ^1^ Translational Research Unit, National Institute for Infectious Diseases Lazzaro Spallanzani-IRCCS, Rome, Italy; ^2^ Unità Operativa Semplice (UOS) Professioni Sanitarie Tecniche, National Institute for Infectious Diseases Lazzaro Spallanzani-IRCCS, Rome, Italy; ^3^ Center for Infectious Disease and Vaccine Research, La Jolla Institute for Immunology, La Jolla, CA, United States; ^4^ Dipartimento di Scienze Biotecnologiche di Base, Cliniche Intensivologiche e Perioperatorie, Università Cattolica del Sacro Cuore, Rome, Italy; ^5^ Diagnostic Labororatory Unit, Mater Olbia Hospital, Olbia, Italy; ^6^ Department of Clinical and Molecular Medicine, “Sapienza” University, S. Andrea University Hospital, Rome, Italy; ^7^ Respiratory Infectious Diseases Unit, National Institute for Infectious Diseases Lazzaro Spallanzani-IRCCS, Rome, Italy

**Keywords:** tuberculosis, rheumatoid arthritis, Th1, antigen-specific response, AIM assay, IFN-γ, MGIA, tuberculosis infection

## Abstract

**Background:**

Subjects with immune-mediated inflammatory diseases (IMID), such as rheumatoid arthritis, with tuberculosis infection (TBI), have a high probability of progressing to tuberculosis disease (TB). We aim to characterize the impact of IMID on the immune response to *M. tuberculosis* (Mtb) in patients with TBI and TB disease.

**Methods:**

We enrolled TBI and TB patients with and without IMID. Peripheral blood mononuclear cells (PBMCs) were stimulated with Mtb-derived epitopes (MTB300). By flow-cytometry, we identified the Mtb-specific CD4^+^ T cells as cytokine-producing T cells or as CD25^+^ CD134^+^ CD4^+^ T cells. Memory and activation status of Mtb-specific T cells were assessed by evaluating: CD153, HLA-DR, CD45RA, CD27. Mycobacterial growth inhibition assay (MGIA) was used to evaluate the ability of PBMCs to inhibit mycobacteria growth. A long-term stimulation assay was used to detect a memory response.

**Results:**

The IMID status and therapy did not affect the magnitude of response to Mtb-antigen stimulation and the number of responders. TBI-IMID showed a cytokine profile like TBI and TB patients. The Mtb response of TBI-IMID patients was characterized by an effector memory and central memory phenotype as in TBI and TB groups. This memory phenotype allowed the increased IFN-γ production after 6 days of MTB300-stimulation. HLA-DR expression on Mtb-specific T cells was associated with TB, whereas CD153 was associated with TBI status. Finally, the TBI-IMID had an MGIA response like TBI and TB patients.

**Conclusion:**

IMID condition does not affect key aspects of the immune response to Mtb, such as the cytokine response, memory and activation profile, and the ability to contain the mycobacteria replication. The immunological characterization of the fragile population of TBI-IMID patients is fundamental to understanding the correlation between protection and disease.

## Introduction

*M. tuberculosis* (Mtb), the etiological agent of tuberculosis (TB), is a leading cause of death from a single infectious agent with an estimated 10.8 million people falling ill with TB in 2023, and an estimated 1.25 million people died (1, 2). It has been estimated that a quarter of the world population has an immune response to Mtb defined as TB infection (TBI) (3). TBI can progress toward TB disease in 5–10% of TBI-infected subjects (4, 5). An immunological balance between the host and Mtb allows the pathogen persistence for years in a quiescent status continuously stimulating the immune system (6). Several conditions could affect this fragile equilibrium leading to a reactivation of Mtb replication and TB disease. Patients with immune-mediated inflammatory diseases (IMID) such as rheumatoid arthritis (RA) might have an increased susceptibility to infections, including TB, because the disease process already compromises their immune system. The risk ranges from 2.0 to 8.9 in RA patients with TBI not receiving IMID therapies and is lower in psoriatic arthritis (PsA), and ankylosing spondylitis (AS) (7–11). The relationship between immunity to Mtb and rheumatic disease is complex, primarily due to immunosuppressive therapies used in the management of IMID. Within the cells of adaptive immunity, T cells, particularly CD4^+^ T cells (helper T cells), play a crucial role in fighting TB (12–16). However, IMIDs, such as RA, are characterized by an aberrant immune response, often involving autoantibodies and dysregulated T-cell responses leading to a higher risk of developing TB disease (8, 9).

Conventional synthetic Disease-Modifying Antirheumatic Drugs (csDMARDs), such as methotrexate, represent the first line immunosuppressive therapy in IMID patients. In patients affected by RA or PsA, biological (b-DMARDs) (17) and targeted synthetic DMARDs (ts-DMARDs) (18), are generally used after csDMARDs failure/intolerance, being highly effective in reducing disease activity and limiting disease progression. Although, this effectiveness can come at the cost of an impaired ability to fight infections (11). TB preventive therapy is mandatory for TBI-IMID patients undergoing treatment with bDMARDs and tsDMARDs such as TNF-α blockers, anti-IL-6, and JAKs inhibitors, considered drugs at high risk of TB reactivation (19). However, the highest TB risk is reported only in patients undergoing therapies with anti-TNF-α having a fourfold risk of developing TB disease (20), due to the known role of TNF-α in granuloma formation and integrity (21). However, following a principle of caution, TB preventive therapy is indicated as well as for other drugs targeting mechanisms of TB immunity (19), significantly down-modulating the immune function, and affecting T cells and macrophage function (7, 9–11) such as JAKs and IL-6 inhibitors.

Therefore, before initiating immunosuppressive therapy, RA patients are screened for TBI using tests such as the tuberculin skin test (TST) or interferon-γ release assays (IGRAs); if either of these tests is positive, a chest X-ray is performed to exclude TB disease (5, 11). If TBI is diagnosed, TB preventive therapy is proposed (7, 22).

In the last years, many studies characterized Mtb immunity to find new correlates of protection to have tools to monitor the immune response for designing TB vaccine. Polyfunctional CD4+ T cells simultaneously producing pro-inflammatory cytokines such as IFN-γ, TNF-α, and IL-2, have been deeply studied as a possible correlate of TB protection without a unique and definitive association with Mtb containment or Mtb replication (23, 24).

Despite this conflicting literature, BCG-based vaccine, the only licensed TB vaccine, and the novel TB vaccine candidates induce polyfunctional CD4^+^ T cells with memory characteristics in both animal models and human studies (23). Therefore, significant focus remains on this specific subset of T cells, as they offer a viable means to assess the memory response induced by vaccines or Mtb infection.

An alternative tool to measure the Mtb-specific immunity is the Activation-Induced Markers (AIM) assay. The simultaneous expression of CD25 and CD134 identified the antigen-specific T cells, as described in response to Mtb-antigen stimulation in HIV-uninfected (25) and HIV-infected individuals (26). The CD134 (OX40) is a member of the TNFR whereas the CD25 is the IL-2 receptor a-chain, these markers are fundamental for survival, proliferation, and cytokine production upon antigen-specific stimulation (25).

Beyond cytokine production, the surface expression of memory and activation markers has been deeply studied. CD153, also known as ‘CD30 ligand’, is a costimulatory molecule member of the TNF superfamily (27). CD4^+^ T cells expressing CD153 in response to Mtb antigens are associated with Mtb protection in both animal and human studies (28). They are inversely associated with the burden of TB disease in humans (29), providing a potential correlate of protection against pulmonary TB disease.

Similarly, other memory and activation markers have also been associated with different TB statuses. The CD27 downregulation (24, 30–32) and HLA-DR upregulation (28, 33–36) on Mtb-specific T cells, are associated with TB disease. Rigorous studies showed that the mycobacterial growth inhibition assay (MGIA) can be used to evaluate vaccine efficacy (37, 38), providing alternative standardized tools to evaluate the ability of the immune response to *in vitro* control the Mtb replication.

Currently, a great effort is underway to develop a new vaccine against TB disease (39–43). Since the TBI-IMID individuals represent an eligible population for TB vaccination, it would be important and clinically relevant to evaluate the status of the immune response to Mtb in these vulnerable subjects at higher risk of developing TB disease. However, few data are available on the Mtb-specific immunity of TBI subjects with IMIDs (42, 44–49).

Based on these premises, we aim to characterize the specific immune response to Mtb antigens in IMID patients with TBI and TB disease evaluating cytokine production, memory and activation markers, and MGIA. A control cohort of TB, TBI, and healthy control (HC) subjects without IMID was included. While TB patients serve as a model for Mtb replication, TBI-IMID subjects represent a model for Mtb containment. Additionally, TBI-IMID subjects are a vulnerable population that can be useful in dissecting the immunologically specific aspects of the TB spectrum.

## Materials and methods

### Study population

This study was approved by the Ethical Committee of the National Institute for Infectious Diseases Lazzaro Spallanzani-IRCCS (approval number 72/2015 and approval number 27/2019). Written informed consent was required to participate in the study. TB patients, TBI subjects and HC were enrolled from 2015 to 2023.

Since TBI in individuals with IMID is associated with a higher risk of progressing to TB disease (7), TB preventive therapy is offered before starting biological therapy. Note that in this study we enrolled 3 patients taking biologic drugs at the time of enrolment and TBI diagnosis. Note that these patients were not screened at the beginning of biologic therapy, although indicated by guidelines (50, 51), and the screening was prescribed only at the time of the therapy switching.

TBI diagnosis was based on a positive score to QuantiFERON (QFT)-Plus assay (Diasorin, Vercelli, Italy) without clinical, microbiological, and radiological evidence of TB disease.

**Figure 1:**
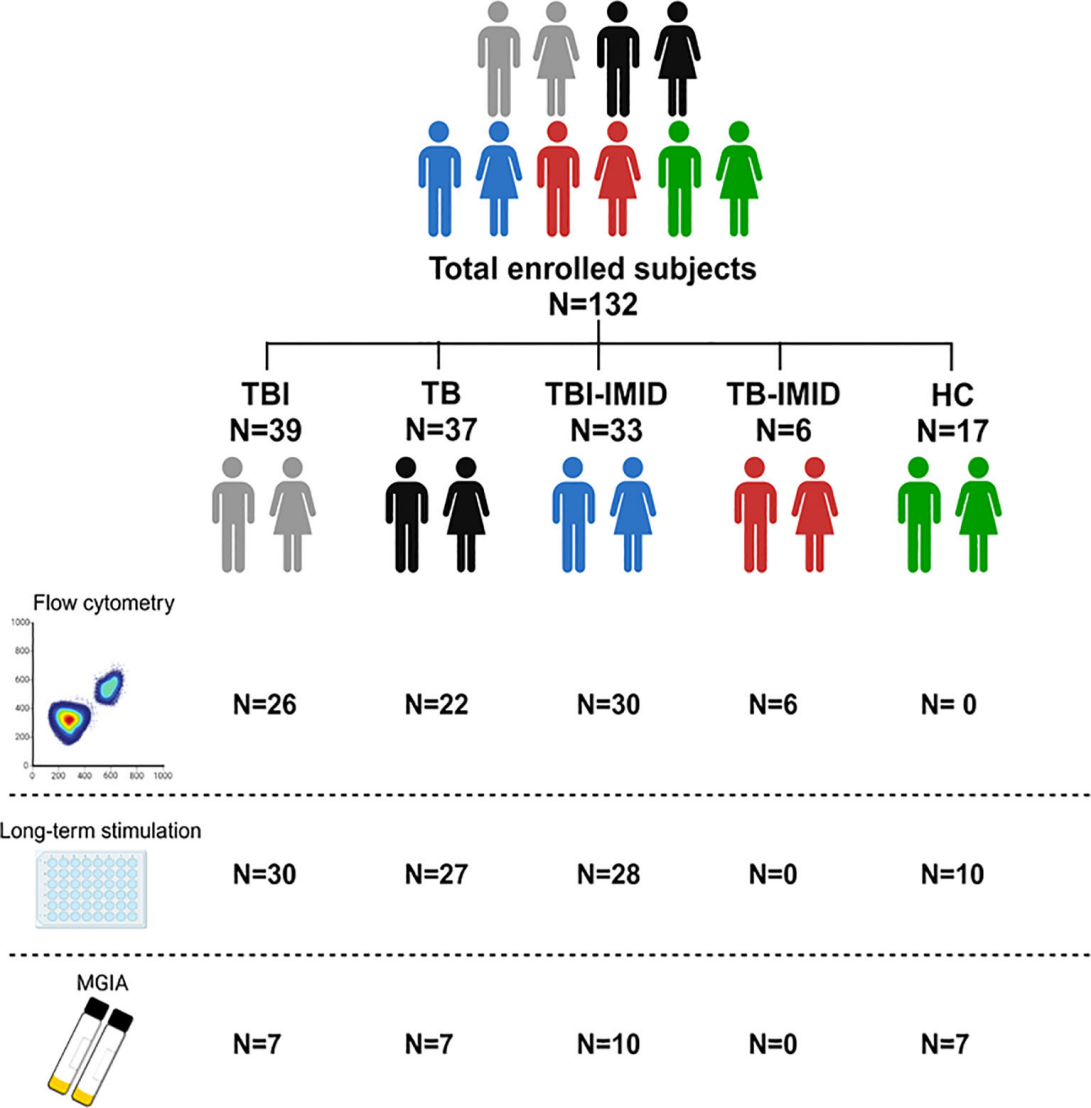
Scheme of subjects enrolled in the study. A total of 132 patients, with different TB statuses and with or without IMID, were enrolled for the study. The number of subjects used for each methodology is reported (Created in BioRender.com).

TBI-IMID patients with a negative response to QFT-Plus showing radiological evidence of scars in the upper lung lobes and reporting a past exposure to TB cases (51), were considered TBI, and preventive therapy was proposed. Among the 9 subjects with TBI-IMID scored negative to QFT-Plus, 2 were taking anti-IL-6 drugs, 2 were taking csDMARDs and corticosteroids, 1 was under csDMARDs and 1 under corticosteroids. TBI and TBI-IMID cohorts were enrolled before starting the TB preventive therapy. TB disease diagnosis was based on microbiological and radiological signs of disease. TB patients were enrolled before starting treatment or within 7 days after therapy initiation. Among the TB-IMID patients, 3 were enrolled between 14 and 40 days of TB therapy and 3 within 7 days of TB therapy. Note that 3 TB-IMID were taking anti-TNF-α drugs at the time of TB diagnosis. Unfortunately, the type of IMID biologic therapy was unknown for one TB-IMID patient. As a control, we enrolled HC who scored negative for QFT-Plus. Demographic and clinical characteristics of all cohorts used in this study are reported in **Table 1** and **Figure 1**. As this is an observational study with unpredictable outcomes, we selected a “convenient sample” of subjects, considering laboratory workflow, enrollment duration, patient flow in the hospital, and experimental protocol costs.

**Table 1:** Clinical characteristics of the enrolled patients.

	TBI	TB	TBI-IMID	TB-IMID	HC	TOTAL	p Value
**N (%)**	39 (29.5)	37 (28)	33 (25)	6 (4.5)	17 (12.9)	132 (100)	
**Age median (IQR)**	45 (32-55)	47 (32-55.5)	58 (48.5-66.5)	51 (41-62.25)	39 (36.5-49.5)	49 (36-57.75)	0.0004*
**Female N (%)**	21 (54)	10 (27)	20 (61)	2 (33)	13 (76.5)	66 (50)	0.0048**
Origin N (%)							
**West Europe**	25 (64)	11 (29.7)	21 (63.6)	2 (33.3)	17 (100)	75 (57.2)	na**
**East Europe**	6 (15.4)	13 (35.1)	5 (15.2)	3 (50)	0 (0)	27 (21)	
**Africa**	3 (7.7)	4 (10.8)	2 (6.1)	1 (16.7)	0 (0)	10 (7.6)	
**Asia**	2 (5)	7 (18.7)	1 (3)	0 (0)	0 (0)	10 (7.6)	
**South America**	3 (7.7)	2 (5.4)	4 (12.1)	0 (0)	0 (0)	9 (6.9)	
**BCG-vaccinated N (%)**	13 (33.3)	25 (83.3)	12 (36.4)	4 (66.7)	0 (0)	54 (41.2)	<0.0001**
Type of IMID N (%)							
**Rheumatoid arthritis**	–	–	20 (60.6)	1 (16.7)	–		**<0.0001 ^§^**
**Psoriatic arthritis**	–	–	10 (30.3)	2 (33.3)	–		na^§§^
**Polymyalgia rheumatica**	–	–	1 (3)	–	–		
**Psoriasis**	–	–	2 (6.1)	1 (16.7)	–		
**Crohn disease**	–	–	–	1 (16.7)	–		
**Ulcerative colitis**	–	–	–	1 (16.7)	–		
**Patients under IMID Therapy N (%)**			20 (61)	5 (83)			0.2857 **
Type of IMID therapy N (%)							
**B**	–	–	3 (15)	2 (40)			0.1183 **^§^**
**B+C**	–	–	–	2 (40)			na ^§§^
**C**	–	–	4 (20)	–			
**cDMARDs**	–	–	4 (20)	1 (20)			
**cDMARDs +/- C +/-**	–	–	9 (45)	–			
QTF-Plus N (%) at the time of enrolment^§^							
**Positive**	39 (100)	23 (76.7)	24 (73)	4 (66.7)	0 (0)	87 (66.4)	na **^‡^**
**Negative**	0 (0)	11 (36.7)	9 (27)	0 (0)	17 (100)	39 (29.8)	
**Indeterminate**	0 (0)	2 (6.7)	0 (0)	1 (16.7)	0 (0)	3 (2.3)	
**Not available**	0 (0)	1 (3.3)	0 (0)	1 (16.7)	0 (0)	2 (1.5)	

N, Number; TBI, TB infection; TB, tuberculosis; IMID, inflammatory mediated immune disease; HC, healthy control; BCG, bacillus Calmette-Guérin; QFT, QuantiFERON; IQR, interquartile range; B, Biological; C, Corticosteroids; cDMARDs, conventional DMARDs; na, not applicable, since Chi-square calculations are only valid when all expected values are greater than 1.0 and at least 20% of the expected values are greater than 5; *Kruskal- Wallis test; **Chi Square test; ^§^Chi Square test among TBI-IMID patients; ^§§^Chi Square test among TB-IMID patients; ^‡^Chi Square test among TB, TBI-IMID and TB-IMID; Significant p values are reported in bold.

To perform this study, we followed the STROBE statement checklist for case-control studies (https://www.strobestatement.org/fileadmin/Strobe/uploads/checklists/STROBE_checklist_v4_case-control.pdf).

### Stimulation and reagents

Withdrawing blood samples were collected in Heparin Blood Collection Tubes (BD Vacutainer^®^ Blood Collection Tubes). Peripheral blood mononuclear cells (PBMCs) were isolated using Ficoll density gradient centrifugation with the SepMate™ tubes (StemCell; Cat.85460) within 4 hours from sampling. Cells were frozen in heat-inactivated fetal bovine serum (FBS) + 10% DMSO and stored in liquid nitrogen until further use. Thawed cells were cultured at a concentration of 0.5-1.0 x 10^6^/mL in 96-multiwell plate for 24 and 48 hours at 37°C, 5% CO_2_ in complete medium [RPMI-1640 (Gibco, CA, USA), 10% fetal bovine serum (FBS) (Gibco, Life Technologies Italia, Monza, Italy), 2mM L-glutamine, and 1% penicillin/streptomycin solution]. PBMCs were stimulated with a pool of 300 Mtb-derived peptides (MTB300, 1.5 µg/ml) (52). MTB300 peptide megapool contained a mixture of 300 Mtb-derived T-cell epitopes from 90 Mtb proteins (including ESAT-6 can CFP10) that target a large fraction of Mtb-specific CD4^+^ T cells, which share epitopes with NTM species (52–54). As positive control cells were stimulated with staphylococcal enterotoxin B (SEB) (Merck Life Science Cat. S4881) at 200ng/mL. The costimulatory monoclonal antibodies α-CD28 and α-CD49 (1µg/mL each) (BD Biosciences, San Jose, USA) were added. BD Golgi Plug was added after 1 hour for cytokine detection, when appropriate. Unstimulated cells were incubated with costimulatory antibodies and Golgi Plug only.

### Intracellular staining assay and flow-cytometry analysis

To characterize the antigen-specific immune response to MTB300, we stained cultured PBMCs after 24h and 48h of incubation. Intracellular staining for cytokine evaluation was performed after 24h of incubation, using: Fixable Viability Stain 700, CD3 V450 (clone UCHT1), CD8 APC-H7 (clone SK1), CD27 BV605 (clone L128), CD45RA PE-Cy7 (clone L48), HLA-DR BV786 (clone G46-6), CD153 PE (R&D System, clone 116614), and CD4 ECD (Beckman Coulter, clone SFCI12T4D11), IFN-γ APC (clone B27), IL2 PerCP-Cy5.5 (clone MQ1-17H12) and TNF-α FITC (clone MAb11) (BD). Brilliant Stain Buffer (BD) and Cytofix/Cytoperm (BD) were used according to the manufacturer’s instructions.

Activation-induced markers (AIM) were evaluated after 48h of incubation (25) using: Fixable Viability Stain 700, CD3 PE-Cy7 (clone SK7), CD8 APC-H7 (clone SK1), CD25 BV480 (clone 2A3), CD27 BV605 (clone L128), CD134 BV421 (clone ACT35), HLA-DR BV786 (clone G46-6) (all from BD), CD153 PE (R&D System, clone 116614) CD4 ECD (Beckman Coulter, clone SFCI12T4D11).

At least 100,000 lymphocytes were acquired using DxFLEX (Beckman Coulter) cytometer. Data were analyzed with FlowJo software (version 10.8.1), PESTLE and SPICE software [provided by Dr. Roederer (version for MacBook, Vaccine Research Center, NIAID, NIH, USA)]. Antigen-specific response was scored positive if the percentage of the stimulated cells were at least 2-fold higher compared to the unstimulated control and if the events gated were at least 10 (see **Supplementary Figure S1** for the gating strategy). The analyses were conducted blindly by two different operators (EP and CF).

### *In vitro* evaluation of the MTB300-specific antigen response after 6 days

The long-term assay was performed in a subgroup of the enrolled subjects with TBI, TB, TBI-IMID, and HC. Thawed PBMCs were seeded at 1x10^6^/mL in a 96-multiwell plate, stimulated with MTB300-peptides (1.5 µg/ml) and cultured for 6 days in a complete medium with α-CD28 and α-CD49 monoclonal antibodies (mAb) (1µg/mL each). At day three, recombinant human IL-2 protein (Bio-techne/R&D System) at 5 IU/mL was added (55). The supernatant was collected after 24h and after 6 days to evaluate the IFN-γ–specific response. The IFN-γ production was evaluated by ELISA assay (Diasorin, Vercelli, Italy) according to the manufacturer’s instructions and the result was expressed as pg/ml (56).

### Mycobacterial growth inhibition assay

The ability of the immune system to inhibit mycobacterial growth was assessed *in vitro* (57). Two million of PBMCs were seeded in 300µl in complete medium, without antibiotics, in a 48-well plate and 300µl of RPMI containing 300 CFU of Bacillus Calmette- Guérin (BCG) Pasteur. Infected PBMCs were incubated for 96 hours at 5% CO_2_. Cells were lysed with sterile water and transferred into the MGIT tube supplemented with 800 µL of PANTA (antibiotics) and OADC broth (Becton Dickinson). MGIT tube were incubated in a Mycobacterial Detection System (BACTEC MGIT 960) until the detection of positivity and growth. As control, the bacterial inoculum used was added directly to a MGIT tube: 300 CFU of BCG Pasteur without added cells were placed directly in the BACTEC MGIT 960 machine on day 0. Data were analyzed as time to positivity (TTP) and expressed in hours, subtracting the TTP of experimental control by the TPP of each experimental condition (ΔTTP).

### Statistical analysis

Data were analyzed using Graph Pad Prism (Version 8.2.1) and SPSS software. The median and interquartile ranges (IQRs) were calculated for continuous measures. For pairwise comparison, Mann–Whitney U and Wilcoxon tests, were used, as appropriate. Friedman’s test was used to compare paired data. Receiver Operator Characteristic (ROC) was used to determine the cut-off values and sensitivity/specificity of long-term stimulation with MTB300 at day 1 and day 6 in TB patients and HC individuals.

## Results

### Characteristics of the population

One hundred and thirty-two individuals with different TB status and with or without IMID, and HC were enrolled. Differences were found for age (p=0.0004) and proportion of females (p=0.0048). About 57% of the enrolled subjects were from Western Europe and 41% were BCG vaccinated. Most TBI-IMID patients had RA (60.6%) and 61% were under immunosuppressive therapy at the time of enrolment. None of TBI-IMID patients developed TB after one year from the end of TB preventive therapy. All the TB-IMID patients were under immunosuppressive therapy (**Table 1**). The characteristics of the specific cohorts for flow-cytometry, long-term and MGIA studies are described in **Supplementary Tables S1**-**S3**.

### Evaluation of Mtb-specific CD4^+^ T cells in TB and TBI subjects with and without IMID

**Figure 2:**
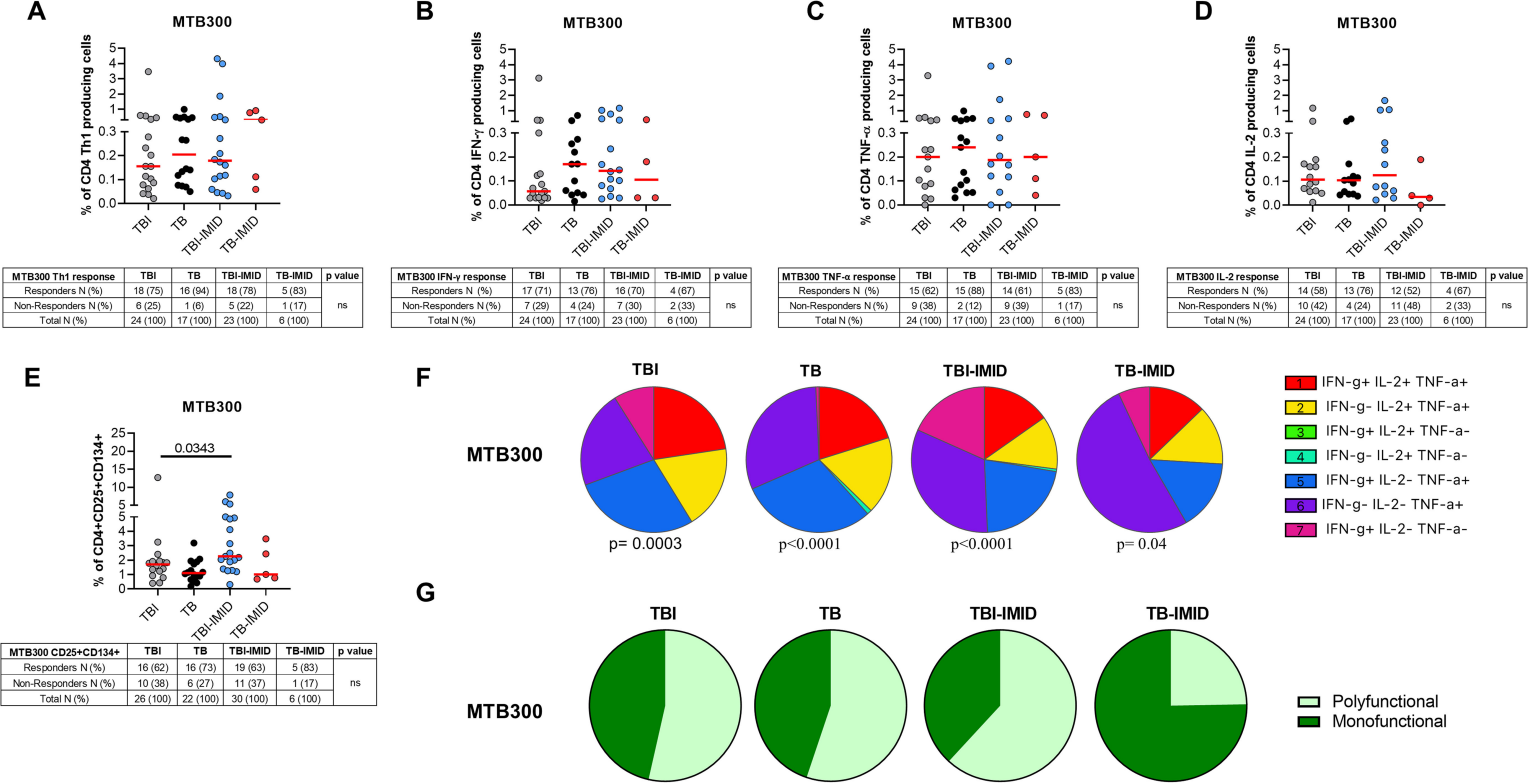
Evaluation of Mtb-specific CD4 T-cell response in TB and TBI subject with and without IMID. PBMCs were stimulated with Mtb-specific antigens (MTB300) for 24 or 48 hours and immune response was evaluated by flow-cytometry. All the analyses were performed only among the responders. **(A)** Antigen-specific response evaluated as total CD4 Th1 cytokine-producing cells after 24 h of stimulation. **(B)** Antigen-specific response evaluated as total IFN-γ^+^ CD4 T cells after 24 h of stimulation. **(C)** Antigen-specific response evaluated as TNF-α^+^ CD4^+^ T cells after 24 h of stimulation. **(D)** Antigen-specific response evaluated as IL-2^+^ CD4^+^ T cells after 24 h of stimulation. **(E)** Antigen-specific response evaluated as CD25^+^ CD134^+^ CD4^+^ T cells after 48h of stimulation. **(A–E)** Tables under the graphs report the number of CD4^+^ T-cell responders to MTB300. Horizontal red lines indicate the median and each dot represents a single subject. Statistical analysis was performed using the Mann-Whitney test. **(F)** Pie charts representing the proportion of different cytokine-producing CD4^+^ T-cell subsets. **(G)** Pie charts representing the proportion of monofunctional and polyfunctional antigen-specific CD4^+^ T cells. **(F, G)** Boolean gate combination and Wilcoxon matched-pairs signed rank test were applied. TB, tuberculosis; TBI, tuberculosis infection; IMID, immune-mediated inflammatory disease; IFN-γ, interferon-gamma; IL-2, interleukine 2; TNF-α, tumor necrosis factor alpha.

To characterize the antigen-specific response, we evaluated both the cytokine-producing T cells and the AIM-positive cells (**Figure 2**, **Supplementary Table S1**). Unfortunately, we did not have sufficient CD8 responders to allow a robust analysis, so we focused only on the CD4^+^ T-cell response. We performed the cytometry study on 26 TBI, 22 TB, 30 TBI-IMID, and 6 TB-IMID individuals (**Supplementary Table S1**). The cytokine-producing cells in response to MTB300 stimulation, hereafter referred to as Th1-response, were similar among groups (**Figure 2A**). Likewise, the frequency of IFN-γ^+^, TNF-α^+^, and IL-2^+^ CD4^+^ T cells was similar among groups (**Figures 2B–D**). Differently, after 48h of incubation, the frequency of antigen-specific CD4^+^ T cells, identified as CD25^+^CD134^+^ (AIM assay), was higher in the TBI-IMID compared to the TBI individuals (p=0.034) (**Figure 2E**). In response to SEB, we observed a comparable distribution of Th1-response among groups and a significantly higher frequency of CD25^+^CD134^+^ CD4^+^ T cells in TBI compared to TB (p= 0.033) and significantly lower frequency compared to the TBI-IMID cohort (p=0.024) (**Supplementary Figures S2A–E**). To better discriminate the effect of IMID therapy on the ability to respond to Mtb stimulation, we stratified the patients according to the type of IMID therapy (**Tables 2**, **3**) Immunosuppressive therapy in the TBI-IMID and TB-IMID cohorts did not have an impact on the number of Mtb responders evaluated as Th1 CD4^+^ T cells and CD25^+^CD134^+^ CD4^+^ T cells (AIM assay) (**Tables 2**, **3**).

**Table 2:** Flow-cytometry study: number of TBI-IMID subjects responding to MTB300-stimulation, stratified according to the IMID therapy.

Type of assay	Enrolled patients according to IMID therapy	Responders N (%)	Non-responders N (%)	p value
**Th1**	**Patients in therapy over total**	11/23 (48)	2/23 (87)	0.6175^§^
	**Patients not in therapy over total**	7/23 (30)	3/23 (13)	
	**Type of therapy over all patients in therapy** **B** **C** **cDMARDs** **cDMARDs +/- C +/-**	0 (0) 3 (27.3) 2 (18.2) 6 (54.5)	1 (50) * 0 (0) 1 (50) 0 (0)	na
**CD25+ CD134+** **(AIM)**	**Patients in therapy over total (%)**	12/30 (40)	6/30 (20)	0.7116^§^
	**Patients not in therapy over total (%)**	7/30 (23)	5/30 (17)	
	**Type of therapy over all patients in therapy** **B** **C** **cDMARDs** **cDMARDs +/- C +/-**	1 (8) * 3 (25) 2 (17) 6 (50)	2 (33) ** 1 (17) 1 (17) 2 (33)	na

N, number; TBI, TB infection; TB, tuberculosis; IMID, inflammatory mediated immune disease; B, Biological; C, Corticosteroids; cDMARDs, conventional DMARDs; AIM, Activation Induced Marker; n, Number; na, not applicable since Chi-square calculations are only valid when all expected values are greater than 1.0 and at least 20% of the expected values are greater than 5; ^§^Fisher’s exact test *anti-CD20; **anti IL-6.

**Table 3:** Flow-cytometry study: number of TB-IMID subjects responding to MTB300-stimulation stratified according to the IMID therapy.

Type of assay	Enrolled patients according to IMID therapy	Responders N (%)	Non- responders N (%)	p value
**Th1**	**Patients in therapy over total**	4/6 (66.6)	1/6 (16.7)	>0.9999^§^
	**Patients not in therapy over total**	1/6 (16.7)	0/6 (0)	
	**Type of therapy over all patients in therapy** **B** **B+C** **cDMARDs**	*2 (40) **1 (20) 1 (20)	0 (0) **1 (100) 0 (0)	n.a
**CD25+ CD134+** **(AIM)**	**Patients in therapy over total**	4/6 (66.6)	1/6 (16.7)	>0.9999^§^
	**Patients not in therapy over total**	1/6 (16.7)	0/6 (0)	
	**Type of therapy over all patients in therapy** **B** **B+C** **cDMARDs**	*2 (40) **1 (20) 1 (20)	0 (0) 1 (100) 0 (0)	na

N, number; TBI, TB infection; TB tuberculosis; IMID, inflammatory mediated immune disease; B, Biological; C, Corticosteroids; cDMARDs, conventional DMARDs; AIM, Activation Induced Marker; n, Number; na, not applicable, since Chi-square calculations are only valid when all expected values are greater than 1.0 and at least 20% of the expected values are greater than 5; ^§^Fisher’s exact test; *one patient was under anti-TNF-α therapy and one patient was under unknown biologic therapy; **patient was under anti-TNF-α therapy and corticosteroids.

### Cytokine profile of Mtb-specific CD4^+^ T cells in TB and TBI subjects with and without IMID

We investigated the functional cytokine profile of Mtb-specific CD4^+^ T cells by applying a boolean gating analysis. All groups were characterized by the presence of IFN-γ^+^ IL-2^+^ TNF-α^+^ CD4^+^ T cells, IFN-γ^-^ IL-2^+^ TNF-α^+^ CD4^+^ T cells, IFN-γ^+^ IL-2^-^ TNF-α^+^ CD4^+^ T cells, IFN-γ^-^ IL-2^-^ TNF-α^+^ CD4^+^ T cells; whereas the IFN-γ^+^ IL-2^-^ TNF-α^-^ CD4^+^ T cells were represented in all groups except for the TB. Note that the TB-IMID showed the highest proportion of IFN-γ^-^ IL-2^-^ TNF-α^+^ CD4^+^ T cells compared to other groups (**Figure 2F**). We then assessed differences by analyzing the total polyfunctional or monofunctional proportion for each group (**Figure 2G**). We observed a similar proportion of polyfunctional and monofunctional Mtb-specific CD4^+^ T cells among TBI, TB, and TB-IMID. Although not significant, TB-IMID patients were characterized by a predominance of monofunctional cytokine-producing CD4^+^ T cells (**Figure 2G**). Moreover, in response to SEB, we found a comparable polyfunctional profile among cohorts with a similar proportion of the different T-cell subsets (**Supplementary Figure S2F**).

### Activation profile of Mtb-specific CD4^+^ T cells in TB and TBI subjects with and without IMID

**Figure 3:**
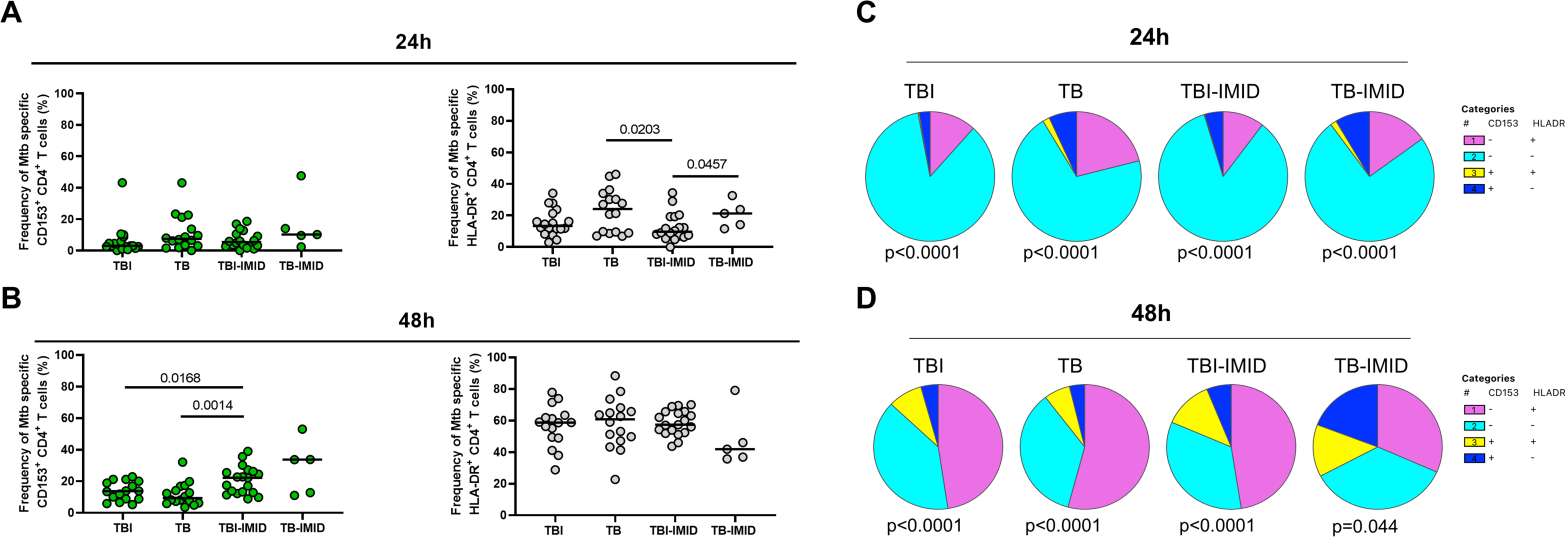
HLA-DR- and CD153 expression on Mtb-specific CD4^+^ T cells in TB and TBI subjects with and without IMID. PBMCs were stimulated with Mtb-specific antigens (MTB300) for 24 hours and 48 hours and the immune response was evaluated by flow-cytometry. The activation profile (CD153^+/-^HLADR^+/-^) of Mtb specific T cells was evaluated only among the responders. **(A)** Activation profile of antigen-specific response, defined as total CD4 Th1 cytokine-producing cells; horizontal black lines indicate the median and each dot represents a single subject. **(B)** Activation profile of antigen-specific response, defined as CD25^+^ CD134^+^ CD4^+^ T- cell response; horizontal lines indicate the median and each dot represents a single subject. **(C)** Pie charts representing the proportion of different CD153^+/-^HLADR^+/-^ CD4^+^ Th1 cytokine-producing cells. **(D)** Pie charts representing the proportion of different CD153^+/-^HLADR^+/-^ CD25^+^ CD134^+^ CD4^+^ T cells. Statistical analysis was performed using the Mann-Whitney test and Wilcoxon test. TB, tuberculosis; TBI, tuberculosis infection; IMID, immune-mediated inflammatory disease; h, hours.

Activation status of Th1-specific CD4^+^ T cells in response to MTB300: we evaluated the surface expression of CD153 and HLA-DR on Th1-specific CD4^+^ T cells after 24h of MTB300-stimulation. TB patients showed a higher frequency of Th1-specific HLA-DR CD4^+^ T cells compared to TBI-IMID (p=0.0203); a similar, but not significant trend, was observed compared to TBI (**Figure 3A**). The HLA-DR expression on Th1 CD4^+^ T cells agrees with previous studies (28). Differently from a previous study (28), the frequency of Th1-specific CD153+ CD4+ T cells was not modulated after 24h of MTB300-stimulation (**Figure 3A**).

Activation status of AIM^+^ CD4^+^ T cells in response to MTB300-stimulation: we evaluated the surface expression of CD153 and HLA-DR on AIM^+^ CD4^+^ T cells after 48h of MTB300-stimulation (**Figure 3B**). In this case, we observed an increased frequency of Mtb-specific CD153^+^ CD4^+^ T cells in TBI-IMID compared to TB (p=0.0014) and in TBI-IMID compared to TBI (p=0.0168). Note that, even if not significant, the median frequency of Mtb-specific CD153^+^ CD4^+^ T cells in TBI individuals was higher than in TB patients. The analysis of the different CD153^+/-^ HLA-DR^+/-^ CD4^+^ T-cell subsets showed an increase of all subsets at 48h at the expense of the CD153^-^ HLA-DR^-^ CD4^+^ T cells (**Figures 3C, D**).

Activation status in response to SEB: we observed an increase of the different CD153^+/-^ HLA-DR^+/-^ CD4^+^ T-cell subsets in response to SEB stimulation compared to the response at 24h and 48h post *in vitro* stimulation (**Supplementary Figure S3**).

**Figure 4:**
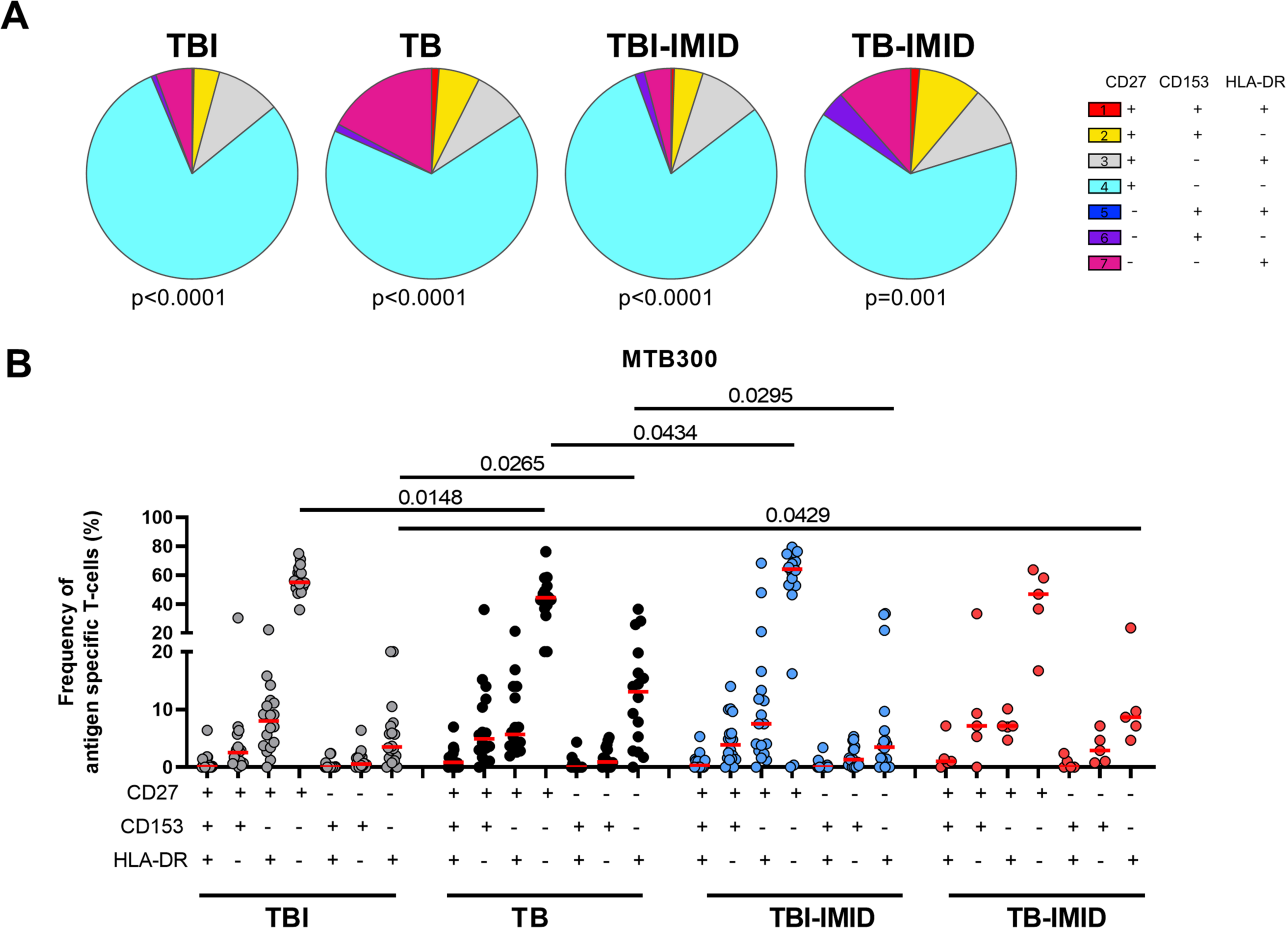
Activation profile of Mtb-specific CD4 Th1 cells in TB and TBI subject with and without IMID. PBMCs were stimulated with Mtb-specific antigens (MTB300) for 24 hours and the immune response was evaluated by flow-cytometry. Antigen-specific response was defined as total CD4 Th1 cytokine-producing cells and the activation profile was evaluated only among the responders. **(A)** Pie charts representing the proportion of CD27^+/-^ CD153^+/-^HLADR^+/-^ CD4 T-cell subsets; Wilcoxon matched-pairs signed rank test were applied. **(B)** Frequency of antigen-specific CD27^+/-^ CD153^+/-^HLADR^+/-^ CD4 T-cell subsets. Horizontal red lines indicate the median and each dot represents a single subject. Statistical analysis was performed using the Mann-Whitney test. TB, tuberculosis; TBI, tuberculosis infection; IMID, immune-mediated inflammatory disease.

CD27, CD153, and HLA-DR evaluation on Th1-specific CD4^+^ T cells in response to MTB300: We evaluated the surface expression of CD27, CD153, and HLA-DR on Th1-specific CD4^+^ T-cells in response to MTB300 (**Figure 4**). The TBI and TBI-IMID subjects had a higher frequency of CD27^+^ CD153^-^ HLA-DR^-^ compared to the TB (p=0.0148 and p=0.0434 respectively). TB patients had a higher frequency of CD27^-^ CD153^-^ HLA-DR^+^ CD4^+^ T cells compared to TBI (p=0.0265) or TBI-IMID (p=0.0295) subjects. Despite the low number of subjects evaluated, the TB-IMID individuals showed an activation profile like the TB patients, with a higher frequency of CD27^-^ CD153^-^ HLA-DR^+^ CD4^+^ T cells compared to TBI (p=0.0429).

CD27, CD153, and HLA-DR evaluation on Th1 CD4^+^ T cells in response to SEB: we found a similar distribution of the activation markers among groups with a prevalent proportion of the CD27^+^ CD153^-^ HLA-DR^-^ CD4^+^ T-cell subset (**Supplementary Figure S4**).

### Memory profile of Mtb-specific CD4 Th1 cells in TB and TBI subjects with and without IMID

**Figure 5:**
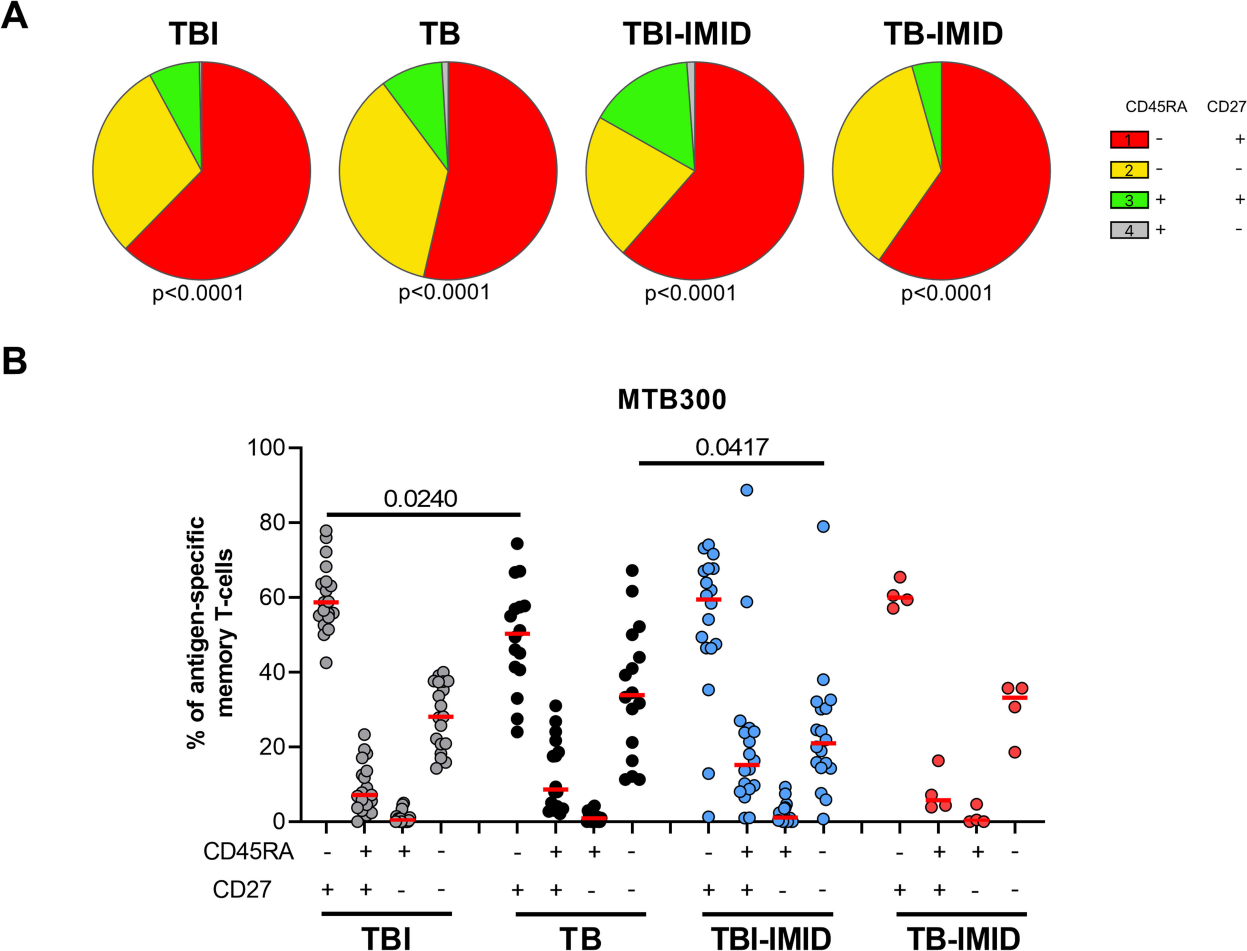
Memory profile of Mtb-specific CD4 Th1 cells in TB and TBI subject with and without IMID. PBMCs were stimulated with Mtb-specific antigens (MTB300) for 24 hours and the immune response was evaluated by flow-cytometry. Antigen-specific response was defined as total CD4 Th1 cytokine-producing cells and the memory profile was evaluated only among the responders. **(A)** Pie charts representing the proportion of CD27^+/-^ CD45RA^+/-^ CD4 T-cell subsets; Wilcoxon matched-pairs signed rank test were applied. **(B)** Frequency of antigen-specific CD27^+/-^ CD45RA^+/-^ CD4 T-cell subsets. Horizontal red lines indicate the median and each dot represents a single subject. Statistical analysis was performed using the Mann-Whitney test. N, number; TB, tuberculosis; TBI, tuberculosis infection; IMID, immune-mediated inflammatory disease.

Then, we investigated if the IMID status may affect the specific response to MTB300, by characterizing the memory profile after 24h of incubation. To have enough events to be analyzed, we evaluated the expression of CD45RA and CD27 within the Mtb-specific T cells producing any Th1 cytokines (IFN-γ, TNF-α, or IL-2). We identified the naïve (N) T cells as CD45RA^+^ CD27^+^, the central memory (CM) as CD45RA^-^CD27^+^, the effector memory (EM) as CD45RA^-^CD27^-^ and the effector (E) as CD45RA^+^CD27^-^ (**Figure 5**) (24). In all groups, independently of the IMID status, the MTB300-specific T cells were mainly CD45RA^-^CD27^+^ (CM) and this subset was significantly higher in the TBI compared to the TB (p=0.024) (**Figure 5B**). Both TB and TB-IMID groups showed an increased frequency of the double negative CD45RA^-^CD27^-^ MTB300-specific CD4 T cells (EM), this difference was significant comparing TB and TBI-IMID subjects (p=0.0417). Note that TBI-IMID individuals showed a high frequency of CD45RA^+^ CD27^+^ T cells (N) compared to the others. In all groups, we found a low frequency of the CD45RA^+^ CD27^-^ T cells (E) (**Figure 5B**). Within each group, as shown by the pie charts, the distribution of the different memory subsets was statistically significant (**Figure 5A**). In response to SEB, we did not find significant differences among groups. However, we found an expansion of the CD45RA^+^CD27^+^ (N) and a reduction of the CD45RA^-^CD27^-^ T cells (EM) compared to the MTB300 response (**Supplementary Figure S5**). Overall, our data indicated that IMID status did not strongly affect the memory profile. Furthermore, we showed that TB patients, regardless of the IMID status, had a higher proportion of Mtb-specific CD45RA^-^CD27^-^ T cells (EM) than TBI subjects, as TB patients downregulated the CD27 expression (30, 58).

### The long-term Mtb stimulation increases the IFN-γ production in subjects with different TB status independently of IMID

**Figure 6:**
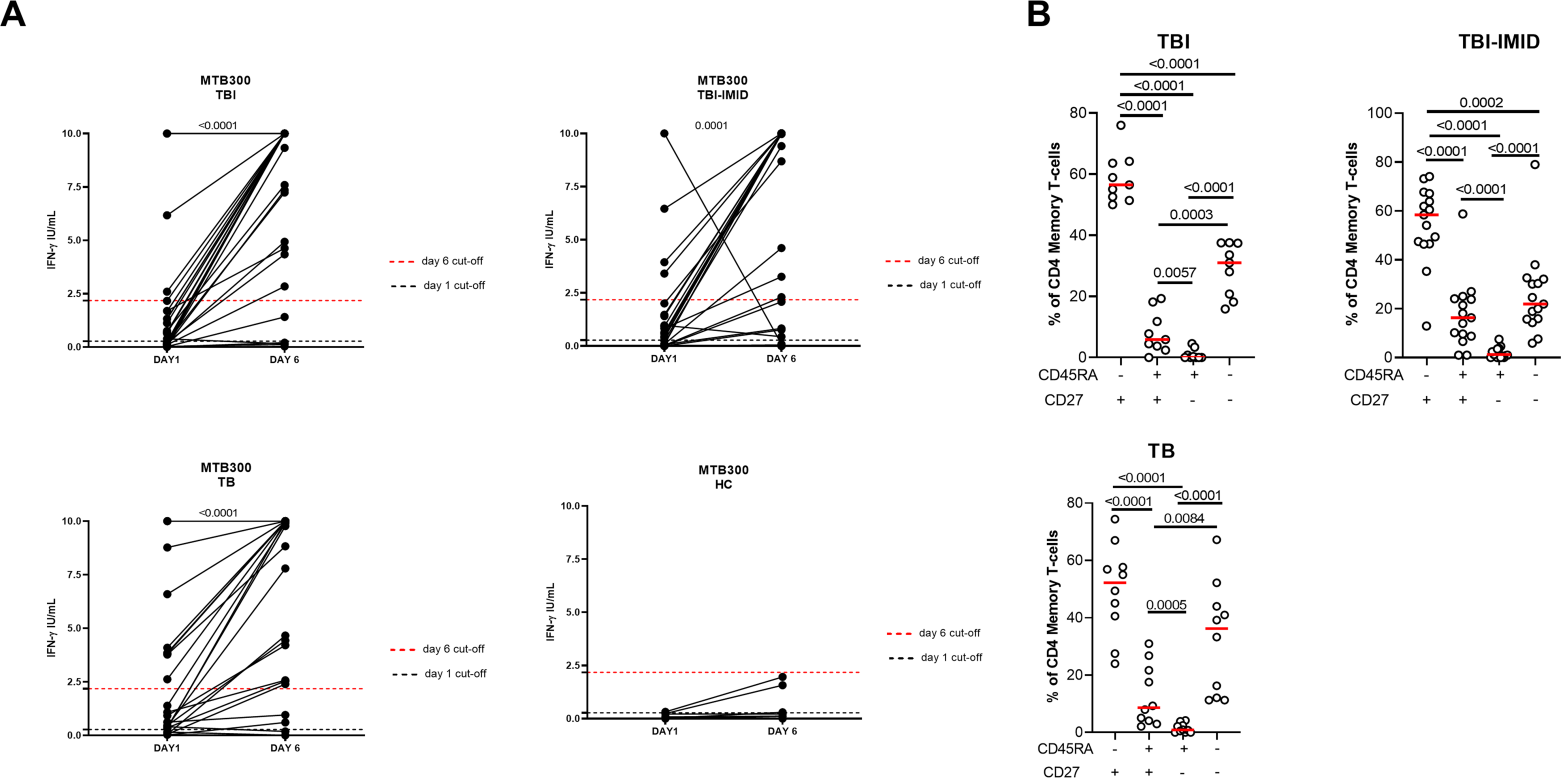
Long-term Mtb stimulation increases the IFN-γ production in subjects with different TB status with and without IMID and healthy controls. **(A)** PBMCs were stimulated with Mtb-specific antigens (MTB300) for 6 days in the presence of IL-2. IFN-γ was evaluated by ELISA on day 1 and day 6 on supernatants. **(B)** Memory profile of Mtb-specific CD4 Th1 cells. PBMCs were stimulated with Mtb-specific antigens (MTB300) for 24 hours and the immune response was evaluated by flow-cytometry. Antigen-specific response was defined as total CD4 Th1 cytokine-producing cells and the activation profile was evaluated only among the responders. The graph represents the frequency of MTB300-specific CD27^+/-^ CD45RA^+/-^ CD4 T-cell subsets of subjects tested in the long-term stimulation assay. Horizontal red lines indicate the median and each dot represents a single subject. Statistical analysis was performed using the Wilcoxon matched-pairs signed rank test. TB, tuberculosis; TBI, tuberculosis infection; IMID, immune-mediated inflammatory disease; HC, healthy control; IFN-γ, interferon-gamma.

We next evaluated if the IMID subjects were able to improve their Mtb-specific T-cell response after 6 days of Mtb antigen stimulation. In a subgroup of individuals (**Supplementary Table S2**), PBMCs were long-term cultured and the Mtb-specific IFN-γ response was evaluated by ELISA. The supernatants were collected after 1 day and after 6 days of incubation (**Figure 6**). The IFN-γ production significantly increased after 6 days of stimulation (TBI: p<0.0001; TBI-IMID: p<0.0001; TB: p<0.0001) (**Figure 6A**). To select a cut-off of the MTB300-stimulation test, we performed a ROC analysis comparing HC and TB patients (day 1: AUC 0.85, p=0.0011; day 6 AUC 0.84, p=0.0016) (**Supplementary Figure S6**). We selected 0.2750 IU/mL as day 1 cut-off (73% sensitivity and 90% specificity) and 2.1750 IU/mL for day 6 (73% sensitivity and 100% specificity). Based on the selected cut-off we found that 46.7% of TBI, 70% of TB, and 50% of TBI-IMID scored positive to MTB300 at day 1, while 73% of TBI, 70% of TB, and 61% of TBI-IMID had a positive response after 6 days of stimulation (day 1 vs day 6: TBI p=0.0350; TB p>0.9999; TBI-IMID p=0.8064) (**Table 4**). Evaluating the rate of change, we found the highest increase in TBI subjects compared to the other groups (**Table 4**).

**Table 4:** Number of responders to *in vitro* long-term stimulation with MTB300.

	Number of responders				
Group	Day 1 (Cut-off=0.275)	Day 6 (Cut-off=2.175)	p*	Rate of change	p**
**TBI [N/total (%)]**	14/30 (46.7)	22/30 (73)	**0.0350**	1036.56	0.0772
**TB [N/total (%)]**	19/27 (70)	19/27 (70)	>0.9999	213.20	
**TBI-IMID [N/total (%)]**	14/28 (50)	17/28 (61)	0.8064	930.66	

N, number; TB, tuberculosis, TBI, tuberculosis infection, IMID, inflammatory mediated immune disease; * Chi-Square test; **Kruskal- Wallis test. Significant p values are reported in bold.

We then investigated whether the ability to retrieve a specific response was related to the memory T cells. We focused on patients with a positive long-term response after 6 days of stimulation and scored positive for an Mtb-specific Th1-response after 24 hours of stimulation. In all groups, we observed a significant predominant memory-subset responsible for the increased IFN-γ production reported at day 6 (p<0.0001) (**Figure 6B**).

### The long-term Mtb stimulation increases the IFN-γ production in TBI-IMID patients scored QFT-plus positive and QFT-plus negative

**Figure 7:**
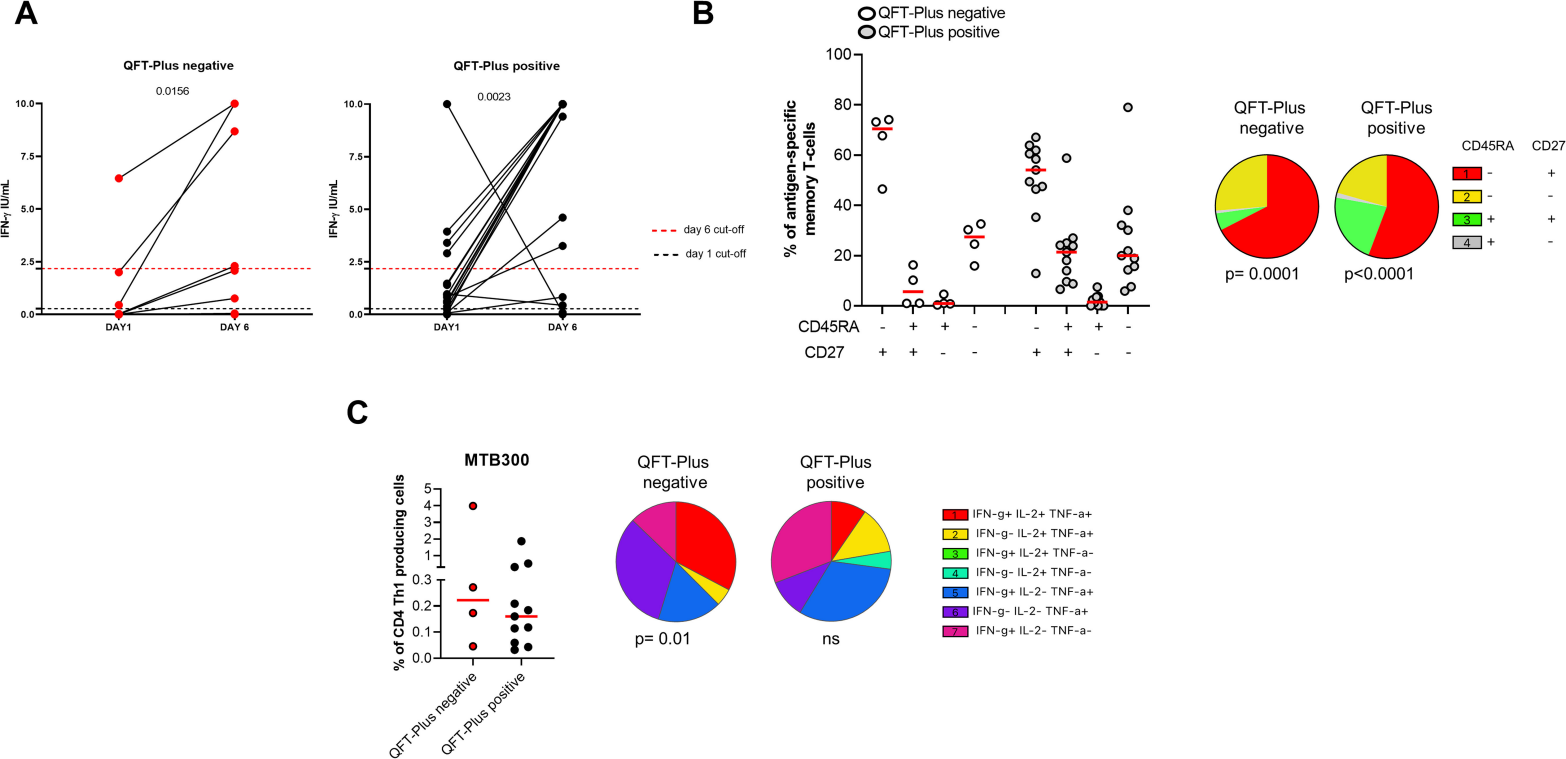
Long-term Mtb stimulation increases the IFN-γ production in TBI-IMID individuals stratified according to the QFT-Plus response. **(A)** PBMCs were stimulated with Mtb-specific antigens (MTB300) for 6 days in the presence of IL-2. IFN-γ was evaluated by ELISA at day 1 and day 6 on supernatants in TBI-IMID QFT-Plus negative and QFT-Plus positive. **(B)** Memory profile of Mtb-specific CD4 Th1 cells. PBMCs were stimulated with Mtb-specific antigens (MTB300) for 24 hours and the immune response was evaluated by flow-cytometry. Antigen-specific response was defined as total CD4 Th1 cytokine-producing cells and the activation profile was evaluated only among the responders. **(C)** Antigen-specific response evaluated as total CD4 Th1 cytokine-producing cells after 24 h of stimulation. Pie charts represent the proportion of different cytokine-producing CD4^+^ T-cell subsets. Horizontal red lines indicate the median and each dot represents a single subject. Statistical analysis was performed using the Mann Whitney test and the Wilcoxon matched pairs signed rank test. TB, tuberculosis; TBI, tuberculosis infection; IMID, immune-mediated inflammatory disease; IFN-γ, interferon-gamma; IL-2, interleukine 2; TNF-α, tumor necrosis factor alpha.

We focused on the TBI-IMID cohort stratifying subjects according to the QFT-Plus results (**Figure 7**, **Supplementary Table S2**). The frequency of IFN-γ production increased in both TB-IMID QFT-Plus positive and QFT-Plus negative (p=0.0156; p=0.0023 respectively). Applying the selected cut-off, at day 6, 4/9 QFT-Plus negative (44%) had a positive long-term response, and 14/21 QFT-Plus positive (67%) had a positive long-term response. Both groups showed a high frequency of CD45RA^-^ CD27^+^ and CD45RA^-^ CD27^-^ Th1-specific CD4 T cells (p=0.0001) (**Figure 7B**). Subjects were also evaluated for their ability to produce Th1-specific cytokines in response to MTB300 (**Figure 7C**). The total Th1-specific response was comparable among groups.

### MGIA response in TB and TBI subjects with and without IMID

**Figure 8:**
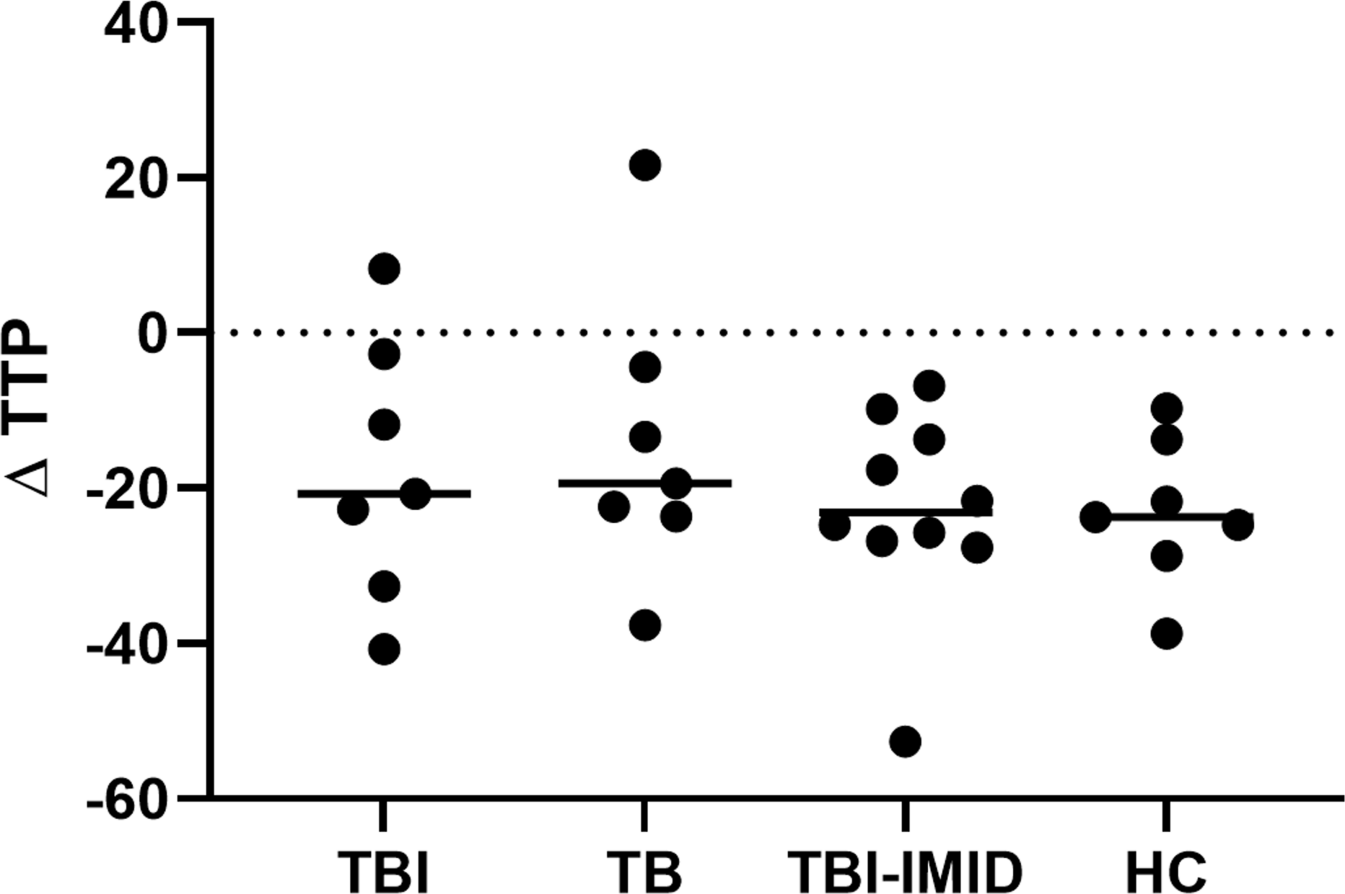
Comparison of MGIA response as TTP in TBI, TB, TBI-IMID, and healthy control individuals. TPP is expressed in hours. As an experimental control, the bacterial inoculum used was added directly to an MGIT tube. The TTP of experimental control was subtracted by the TPP of each experimental condition. Data are represented as the median. Mann-Whitney test was applied. TB, tuberculosis, TBI, TB infection; TTP, time to positivity; HC, healthy control.

To evaluate if the IMID status impaired the ability of PBMCs to control mycobacteria replication, we used the MGIA, using the vaccine strain BCG, in a subgroup of individuals with different TB statuses and with or without IMID (**Figure 8**). Clinical and demographical characteristics are reported in **Supplementary Table S3**. The MGIA response, expressed as the time to positivity (TTP) (59), was compared among different groups (**Figure 8**). No TTP differences were observed among groups, indicating that TBI-IMID could control the mycobacteria replication like non-IMID individuals.

## Discussion

### Summary

In this study, we showed that the memory and activation status of individuals with TBI-IMID are similar to those of patients with TB or TBI without IMID. This finding suggests that TBI-IMID individuals possess effective Mtb-specific immunity and can control mycobacterial replication, as assessed by MGIA, just like non-IMID subjects. Additionally, TB-IMID patients exhibit a cytokine response and activation profile similar to TBI individuals without IMID. Thus, other immunological mechanisms might account for the impaired Mtb-specific immunity in this susceptible IMID group.

### Mtb-specific T cells

To evaluate Mtb-specific immunity besides the response to ESAT-6 and CFP-10 (RD1 antigens) evaluated by QFT-Plus, we used a different Mtb-peptide pool, the “MTB300-peptide- megapool” containing a mixture of 300 Mtb-derived T-cell epitopes from 90 Mtb proteins (including ESAT-6 can CFP10) targeting a large fraction of Mtb-specific CD4+ T cells and sharing epitopes with NTM species (52–54). All groups’ patients respond to MTB300-stimulation in terms of cytokine-production or AIM^+^ CD4^+^ T cells. TB therapy reduces the Mtb load in the host, leading to a decreased Mtb-specific immune response (60–62). In our experimental setting, half of TB-IMID patients have been enrolled during TB therapy. Although a limited number of patients enrolled, the TB-IMID cohort showed a similar level of cytokine-producing CD4^+^ T cells or AIM^+^ CD4^+^ T cells compared to TB individuals enrolled before starting the TB therapy.

All groups’ patients, independently of IMID and TB status, had polyfunctional CD4^+^ T cells producing at least two cytokines and monofunctional CD4^+^ T cells for IFN-γ or TNF-α as previously demonstrated for MTB300 (52). According to the literature, the polyfunctional response is associated with infection control in the case of HIV and *L.major* infections (63, 64), whereas controversial data are available for TB (23). In our study, TBI-IMID showed a cytokine profile like TBI and TB patients, indicating that the Mtb-specific cytokine response is not altered by the IMID status. Differently, TBI individuals living with HIV infection had a lower percentage of cytokine-producing Mtb-specific CD4^+^ T cells and a decrease of the double positive IFN-γ^+^ IL-2^−^ TNF-α^+^ CD4^+^ T cells (65). These results indicate different mechanisms characterizing the groups at high TB risk such as the IMID subjects and the people living with HIV. A higher polyfunctional response associated with the TBI-IMID status and a monofunctional response characterized the TB-IMID patients, suggesting a loss of polyfunctional CD4^+^ T-cell response in TB disease. Since half of TB-IMID patients have been enrolled during TB therapy, this status may lead to a monofunctional response switching. Our recent findings support these data, indicating that TB therapy did not significantly impact the cytokine response in TBI-IMID. However, it did result in a notable reduction of triple functional CD4 T cells in both TBI subjects and TB patients (44). Despite the small size of the TB-IMID cohort, we present these findings as describing such a rare group warrants further research to explore any potential link between Mtb-specific responses and TB outcomes. Lastly, we believe these findings could aid in developing new correlates of protection for patients with varying TB statuses.

According to the literature, the antigen-specific response can be investigated by the co-expression of CD25 and CD134 through the AIM assay (25). Our results indicated that IMID-therapy does not affect the count of either cytokine or AIM responders. The AIM assay reveals a stronger immune response than the intracellular cytokine assay, making the differences between groups more evident. This finding is supported by an earlier study that demonstrated the AIM assay’s superior capability in detecting antigen-specific T cells compared to cytokine-producing T cells (66).

### Memory status of Mtb-specific CD4^+^ T cells

Given the conflicting data on the relationship between cytokine profiles and TB protection, other elements of the immune response, such as the memory and activation status of antigen-specific T cells, were evaluated.

Here we showed that TBI-IMID patients were characterized by an Mtb-specific memory response CD45RA^-^ CD27^+/-^, as previously described in TB and TBI individuals (24, 67–69). In particular, the Mtb-specific CD45RA^-^ CD27^–^ CD4^+^ T cells (effector memory) characterized the TB patients, whereas the Mtb-specific CD45RA^-^ CD27^+^ CD4^+^ T cells (central memory) characterized the TBI individuals. These results highlight the role of the different T-cell subsets contrasting the Mtb replication. The presence of Mtb at a low replication rate during the TBI status constantly stimulated a central memory response fundamental to containing the Mtb load. Vice versa, during the TB disease, the frequency of Mtb-specific effector memory T cells increases to actively contrast the Mtb replication. These findings are consistent with earlier studies on patients with varied TB status, both with and without IMID (24, 44), and those with HIV (67).

The memory phenotype is fundamental for the increased IFN-γ-specific response after MTB300-long-term stimulation. It has been demonstrated that TBI-IMID subjects have a low IFN-γ response to QFT-Plus and a high proportion of results in the uncertain range (70). The long-term stimulation allows the recovery of the Mtb-specific response in TBI-IMID scored negative to QFT-Plus, suggesting this approach as an alternative diagnostic tool in this category of TBI subjects. Note that TBI-IMID had a high proportion of Mtb-specific CD45RA^+^ CD27^+^ CD4^+^ T cells and this proportion was lower in TBI-IMID QFT-Plus negative compared to QFT-Plus positive. Since the CD45RA^+^ CD27^+^ T-cell subset could contain the stem cell memory T cells CD45RA^+^ CD27^+^ CCR7^+^, as already demonstrated in TBI individuals (71), this subset may play a role in our TBI-IMID cohort modifying the phenotype of Mtb-specific T cells.

### Activation status of Mtb specific CD4^+^ T cells

Studies in animal models have demonstrated that Mtb-specific CD4 T cells expressing CD153 are protective against Mtb infection (28, 29, 72, 73). Moreover, the Mtb-specific CD153^+^ CD4^+^ T cells are inversely proportional to bacterial load and TB severity in patients with TB disease (29). In this study, we observed an increased CD153 expression only after 48 hours of MTB300-stimulation in TBI-IMID and TBI individuals. The TBI-IMID showed an increase of Mtb-specific CD153^+^ CD4^+^ T cells compared to TBI, supporting the role of autoimmunity in the CD153-expression (74). Age could be an important factor influencing this result, considering that our study population was older (**Table 2**) than the cohorts of previous studies describing the CD153-expression on Mtb-specific T cells (28, 29, 33). Since the expression of CD153 has been associated with senescence-associated T-cell impairment (75), we retain that age could be an important factor influencing this result. Based on this evidence, probably other mechanisms associated with immune-senescence affected the CD153 expression on Mtb-specific CD4^+^ T cells. Also, the origin of our patients may have had an impact on CD153 expression. Our TBI population was mainly from low TB endemic countries, whereas the previous studies were performed in Africa (28, 29, 33), where repeated exposures to Mtb could affect the Mtb-immunity and modify the activation status.

The expression of HLA-DR on Mtb-specific T cells is increased in TB after 24 hours of MTB300-stimulation, as previously shown (28, 33, 34). Moreover, the HLA-DR expression increased in all groups after 48 hours of Mtb stimulation indicating a time-dependent differentiation of CD4^+^ T cells (66). The TB-IMID patients seem to have a high proportion of activated Mtb-specific HLA-DR^+^ CD4^+^ T cells, like TB patients. Differently, in TBI-IMID, the proportion of Mtb-specific HLA-DR^+^ CD4^+^ T cells is like TBI, demonstrating a comparable activation status independent of the IMID comorbidity. Interestingly, the expression of HLA-DR and CD27 on Mtb-specific CD4^+^ T cells demonstrated a dichotomous profile: the Mtb-specific CD27^-^ CD153^-^ HLA-DR^+^ CD4^+^ T cells associated with TB status and the Mtb-specific CD27^+^ CD153^-^ HLA^-^ DR^-^ CD4^+^ T cells associated to TBI status, indicating a highly differentiated profile in patients with TB (24).

### Mycobacterial growth inhibition assay

In this study we aimed to characterize the Mtb-specific immunity, however, we included the MGIA experiment to have a global evaluation of the immune defense against mycobacteria, looking simultaneously at the innate and adaptive compartments. Monocyte subsets are key cells of the innate immune response and are fundamental to stimulating adaptive immunity acting as antigen-presenting cells. It has been demonstrated that the monocytes/lymphocytes (M/L) ratio has a predictive value for TB disease, in particular, a high M/L is associated with the TB disease (76). MGIA is a largely used test to monitor the vaccine response and a powerful tool to indirectly test the presence of functional T cells and competence of the innate compartment (37, 38). Our results demonstrated that TBI-IMID had the same ability as TBI and TB to contain the mycobacteria replication evaluated by MGIA.

### Translational application of the study

Since the BCG-based vaccine is the only TB vaccine licensed and has a low efficacy for protecting adolescents and adults from TB disease, the development of new vaccine strategies is the main goal of the TB control programs (77). The fragile population of TBI-IMID patients is a potential target of the new vaccine approach, and its immunological characterization is fundamental to develop new biomarkers of protection and disease and to develop alternative therapeutic strategies based on host-direct therapy (78). Since few studies are available on Mtb-immunity in IMID patients (44, 48, 70), we contributed to build the immunological story characterizing the TBI-IMID subjects.

### Limits of the study and future directions

The non-homogeneous IMID cohort may limit the solidity of data, however, the different types of ongoing IMID-therapy did not have an impact on the number of responders, as previously shown (44, 70). Due to the availability of cells, not all patients were characterized using the same methodology. Nonetheless, the statistically significant differences observed among the groups validate the consistency of our results. The immunological scenario of IMID is large and complex, however, we tried to summarize a particular aspect of the disease that increases the risk of TB development. Larger studies are needed to elucidate other features of the Mtb-induced immunity. In particular the investigation of specific mechanisms that increase the TB risk in patients undergoing therapy such as IL-6 or JAK inhibitors (19). Although the number of TB-IMID patients enrolled was low, we included this population because it was an opportunity to study Mtb immunity in a rare cohort, larger studies are necessary to eventually identify a correlation between the Mtb-specific immune response and TB outcome. Since TBI-IMID subjects had remote exposure to Mtb and TBI had a recent TB contact, future studies should include TBI with a remote TB exposure. It is known that only 5% of TBI individuals progress to TB disease within the first 5 years from the TB contact (79), whereas only 2% evolve to TB disease during their lifetime (80). Studying TBI individuals with different statuses of TB exposure is a useful approach to understand which type of immunity promotes the eradication or the efficient containment of Mtb. In low TB-endemic countries, TBI patients with remote exposure are not eligible for TB preventive therapy (22) and therefore the evaluation over time of their Mtb immunity could contribute to describing the mechanisms of Mtb containment.

Besides these limitations, we deeply characterized the immune response to Mtb of patients with different TB status in the presence or absence of IMID, showing that IMID status did not affect the Mtb immunity. Recently, it has been demonstrated that Mtb DNA can be detected in PBMC from subjects with TBI and TB disease. In particular, the CD34^+^ cells represent a niche for Mtb (81). This approach could be applied to immunological studies to find a correlation between the Mtb-specific immune response and the presence of Mtb in the circulating reservoir.

### Conclusions

We added a contribution to the knowledge of Mtb immunity in this fragile cohort to TBI-IMID subjects demonstrating that TB-IMID patients had a cytokine response, a memory and activation profile, and an ability to contain the mycobacteria replication similar to TBI individuals without IMID. The immune tools available do not completely explain the mechanisms of impairment of Mtb-specific immunity in this vulnerable population and new approaches are needed to overcome this limitation.

## Data Availability

The datasets presented in this study can be found in online repositories. The names of the repository/repositories and accession number(s) can be found below: The raw data are available in our institutional repository (rawdata.inmi.it), subject to registration. The data can be found by selecting the article of interest from a list of articles ordered by year of publication. No charge for granting access to data is required. In the event of a malfunction of the application, the request can be sent directly by e-mail to the Library (biblioteca@inmi.it).
