# Supplementary material for: Specific immune response to M. tuberculosis and ability to in vitro control mycobacterial replication are not impaired in subjects with immune-mediated inflammatory disease and tuberculosis infection
Source: Front Immunol. 2025 Jan 13;15:1484143. doi: 10.3389/fimmu.2024.1484143 (PMC11770028; doi:10.3389/fimmu.2024.1484143)
Supplement: Supplementary file 1 [file DataSheet1.docx]

Supplementary Material

# Supplementary Tables

**Supplementary Table S1**

| **Supplementary Table S1: Clinical Characteristics of the enrolled patients whose samples were used or the flow cytometry analysis** | | | | | | | |  |
| --- | --- | --- | --- | --- | --- | --- | --- | --- |
|  | **TBI** | **TB** | **TBI-IMID** | **TB-IMID** | **TOTAL** | ***p* Value** | |  |
| **N (%)** | 26 (31) | 22 (26.2) | 30 (35.7) | 6 (7.1) | 84 (100) |  | |  |
| **Age median (IQR)** | 42  (28-60) | 50  (35-58) | 61  (49-67) | 52  (41-62) | 52  (37-64) | **0.0084*** | |  |
| **Female N (%)** | 14 (54) | 7 (32) | 19 (45) | 2 (33) | 42 (50) | 0.12** | |  |
| **Origin N (%)** |  |  |  |  |  | na** | |  |
| **West Europe** | 16 (61.6) | 9 (41) | 20 (66.7) | 2 (33) | 47 (57) |  |  |  |
| **East Europe** | 5 (19) | 8 (36) | 4 (13.3) | 3 (50) | 19 (23) |  |  |  |
| **Asia** | 2 (7.7) | 0 (0) | 2 (6.7) | 1 (17) | 5 (6) |  |  |  |
| **Africa** | 2 (7.7) | 4 (18) | 0 (0) | 0 (0) | 6 (7) |  |  |  |
| **South America** | 1(4) | 1 (5) | 4 (13.3) | 0 (0) | 6 (7) |  |  |  |
| **BCG-vaccinated N (%)** | 10 (38) | 12 (54.5) | 10 (33.3) | 4 (67) | 36 (43) | 0.26** | |  |
| **Type of IMID N (%)** |  |  |  |  |  |  | |  |
| **Rheumatoid arthritis** | - | - | 19 (63.3) | 1 (16.7) |  | **<0.0001 ^§^** | |  |
| **Psoriatic arthritis** | - | - | 9 (30) | 2 (33.3) |  | na ^§§^ | |  |
| **Polymyalgia rheumatica** | - | - | 1 (3.3) | - |  |  | |  |
| **Psoriasis** | - | - | 1 (3.3) | 1 (16.7) |  |  | |  |
| **Crohn disease** | - | - | - | 1 (16.7) |  |  | |  |
| **Ulcerative colitis** | - | - | - | 1 (16.7) |  |  | |  |
| **Patients in IMID therapy N (%)** |  |  | 18 (60) | 5 (83) |  | 0.2774** |  |  |
| **Type of IMID therapy N (%)** |  |  |  |  |  |  | |  |
| **B** | - | - | 3 (17) | 2 (40) |  | 0.2173 ^§^ | |  |
| **B+C** | - | - | - | 2 (40) |  |  | |  |
| **C** | - | - | 4 (22) | - |  | na ^§§^ | |  |
| cDMARDs | - | - | 3 (17) | 1 (20) |  |  | |  |
| cDMARDs **+/- C +/-** | - | - | 8 (44) | - |  |  | |  |
| **QTF-Plus N (%) at the time of enrolment** |  |  |  |  |  |  | |  |
| **Positive** | 26 (100) | 14 (63.6) | 19 (63.3) | 4 (66.7) | 63 (76) |  | |  |
| **Negative** | 0 (0) | 7 (32) | 11 (36.7) | 0 (0) | 17 (20) | na^ǂ^ | |  |
| **Indeterminate** | 0 (0) | 1 (4.5) | 0 (0) | 1 (16.7) | 2 (2) |  | |  |
| **Not available** | 0 (0) | 0 (0) | 0 (0) | 1 (16.7) | 1 (1) |  | |  |
| **Footnotes**: N: Number; HC: healthy control; TBI: TB infection; TB: tuberculosis; IMID: inflammatory mediated immune disease; BCG: bacillus Calmette-Guérin; QFT: QuantiFERON; IQR: interquartile range; B: Biological; C: Corticosteroids; cDMARDs: conventional DMARDs; na: not applicable, since Chi-square calculations are only valid when all expected values are greater than 1.0 and at least 20% of the expected values are greater than 5; *Kruskal- Wallis test; **Chi Square test; §: Chi Square test among TBI-IMID patients; §§: Chi Square test among TB-IMID patients; ǂ Chi Square test among TB, TBI-IMID and TB-IMID; Significant p values are reported in bold. | | | | | | | | |

**Supplementary Table S2**

| **Supplementary Table S2: Clinical Characteristics of the enrolled patients used for the long-term stimulation** | | | | | | |  |
| --- | --- | --- | --- | --- | --- | --- | --- |
|  | **TBI** | **TB** | **TBI-IMID** | **HC** | **TOTAL** | ***p* Value** |  |
| **N (%)** | 30 | 27 | 28 | 10 | 95 |  |  |
| **Age median (IQR)** | 48 (32.8-57) | 45 (32-56) | 56 (48-64) | 37 (28.8-64) | 48 (35-60) | **0.0006*** |  |
| **Female N (%)** | 15 (50) | 10 (37) | 19 (67.85) | 7 (70) | 33 (34.7) | 0.0894** |  |
| **Origin N (%)** |  |  |  |  |  | na** |  |
| **West Europe** | 21 (70) | 9 (33.33) | 16 (57.14) | 10 (100) | 55 (57.9) |  |  |
| **East Europe** | 5 (16.66) | 9 (33.33) | 5 (17.86) | 0 (0) | 19 (20) |  |  |
| **Asia** | 2 (6.66) | 2 (7.4) | 2 (7.14) | 0 (0) | 6 (6.3) |  |  |
| **Africa** | 0 (0) | 5 (18.5) | 1 (3.6) | 0 (0) | 6 (6.3) |  |  |
| **South America** | 2 (6.66) | 2 (7.4) | 4 (14.3) | 0 (0) | 9 (9.5) |  |  |
| **BCG vaccinated N (%)** | 8 (26.66) | 17 (62.96) | 12 (42.85) | 0 (0) | 38 (40) | **0.0074**** |  |
| **Type of IMID N (%)** |  |  |  |  |  |  |  |
| **Rheumatoid arthritis** | - | - | 17 (60.7) | - |  |  |  |
| **Psoriatic arthritis** | - | - | 9 (32.1) | - |  | **<0.0001^§^** |  |
| **Polymyalgia rheumatica** | - | - | 1 (3.6) | - |  |  |  |
| **Psoriasis** | - | - | 1 (3.6) | - |  |  |  |
| **Patients in IMID therapy N (%)** | - | - | 16 (57) | - |  |  |  |
| **Type of IMID therapy** |  |  |  |  |  |  |  |
| **B** | - | - | 3 (19) | - |  |  |  |
| **C** | - | - | 2 (12) | - |  | 0.062^§^ |  |
| **cDMARDs** | - | - | 3 (19) | - |  |  |  |
| **cDMARDs +/- C +/-** | - | - | 8 (50) | - |  |  |  |
| **QTF Plus N (%) at the time of enrolment** |  |  |  |  |  | 0.3231 ǂ |  |
| **Positive** | 30 (100) | 19 (70.4) | 19 (67.85) | 0 (0) |  |  |  |
| **Negative** | 0 (0) | 7 (25.9) | 9 (32.14) | 10 (100) |  |  |  |
| **Not avaiable** | - | 1 (3.7) | - | - |  |  |  |
| **Footnotes**: N: Number; HC: healthy control; TBI: TB infection; TB: tuberculosis; IMID: inflammatory mediated immune disease; BCG: bacillus Calmette-Guérin; QFT: QuantiFERON; IQR: interquartile range; B: Biological; C: Corticosteroids; cDMARDs: conventional DMARDs; na: not applicable, since Chi-square calculations are only valid when all expected values are greater than 1.0 and at least 20% of the expected values are greater than 5; *Kruskal- Wallis test; **Chi Square test; §: Chi Square test among TBI-IMID patients; ǂ Chi Square test among TB, TBI-IMID; Significant p values are reported in bold | | | | | | | |

**Supplementary Table S3**

| **Supplementary Table S3: Clinical Characteristics of the enrolled patients whose samples were used for the MGIA.** | | | | | | |  |
| --- | --- | --- | --- | --- | --- | --- | --- |
|  | **TBI** | **TB** | **TBI-IMID** | **HC** | **TOTAL** | ***p* Value** |  |
| **N (%)** | 7 (22.6) | 7 (22.6) | 10 (32.2) | 7 (22.6) | 31 (100) |  |  |
| **Age median (IQR)** | 48 (23-73) | 44 (31-44) | 58 (42.75-64.75) | 46 (31-50) | 48 (35-63) | 0.2235* |  |
| **Female N (%)** | 3 (43) | 3 (43) | 6 (60) | 5 (71) | 17 (55) | 0.6376** |  |
| **Origin N (%)** |  |  |  |  |  | na** |  |
| **West Europe** | 4 (57) | 2 (29) | 6 (60) | 7 (100) | 19 (61.2) |  |  |
| **East Europe** | 2 (29) | 3 (43) | 3 (30) | 0 (0) | 8 (26) |  |  |
| **Asia** | 1 (14) | 0 (0) | 0 (0) | 0 (0) | 1 (3.2) |  |  |
| **Africa** | 0 (0) | 1 (14) | 0 (0) | 0 (0) | 1 (3.2) |  |  |
| **South America** | 0 (0) | 1 (14) | 1 (10) | 0 (0) | 2 (6.4) |  |  |
| **BCG vaccinated N (%)** | 3 (43) | 4 (57) | 4 (40) | 0 (0) | 11 (35) | 0.1363** |  |
| **Type of IMID N (%)** |  |  |  |  |  |  |  |
| **Rheumatoid arthritis** | - | - | 6 (60) | - |  | 0.0578 § |  |
| **Psoriatic arthritis** | - | - | 3 (30) | - |  |  |  |
| **Polymyalgia rheumatica** | - | - | 1 (10) | - |  |  |  |
| **Patients in IMID therapy N (%)** | - | - | 7 (70) | - | 9 (90) |  |  |
| **Type of IMID therapy** |  |  |  |  |  | 0.8300§ |  |
| **B** | - | - | 2 (20) | - |  |  |  |
| **cDMARDs** | - | - | 2 (20) | - |  |  |  |
| **cDMARDs +/- C +/-** | - | - | 3 (30) | - |  |  |  |
| **QTF Plus N (%) at the time of enrolment** |  |  |  |  |  | 0.5851 ǂ |  |
| **Positive** | 7 (100) | 4 (57) | 7 (70) | 7 (100) | 25 (80.6) |  |  |
| **Negative** | 0 (0) | 3 (43) | 3 (30) | 0 (0) | 6 (19.4) |  |  |
| **Footnotes:** N: Number; HC: healthy control; TBI: TB infection; TB: tuberculosis; IMID: inflammatory mediated immune disease; BCG: bacillus Calmette-Guérin; QFT: QuantiFERON; IQR: interquartile range; I: Immuno-suppressant; B: Biological; C: Corticosteroids; cDMARDs: conventional DMARDs; na: not applicable, since Chi-square calculations are only valid when all expected values are greater than 1.0 and at least 20% of the expected values are greater than 5; *Kruskal- Wallis test; **Chi Square test; §: Chi Square test among TBI-IMID patients; ǂ Chi Square test among TB, TBI-IMID; Significant p values are reported in bold. | | | | | | | |

# Supplementary Figures

**Supplementary Figure S1**


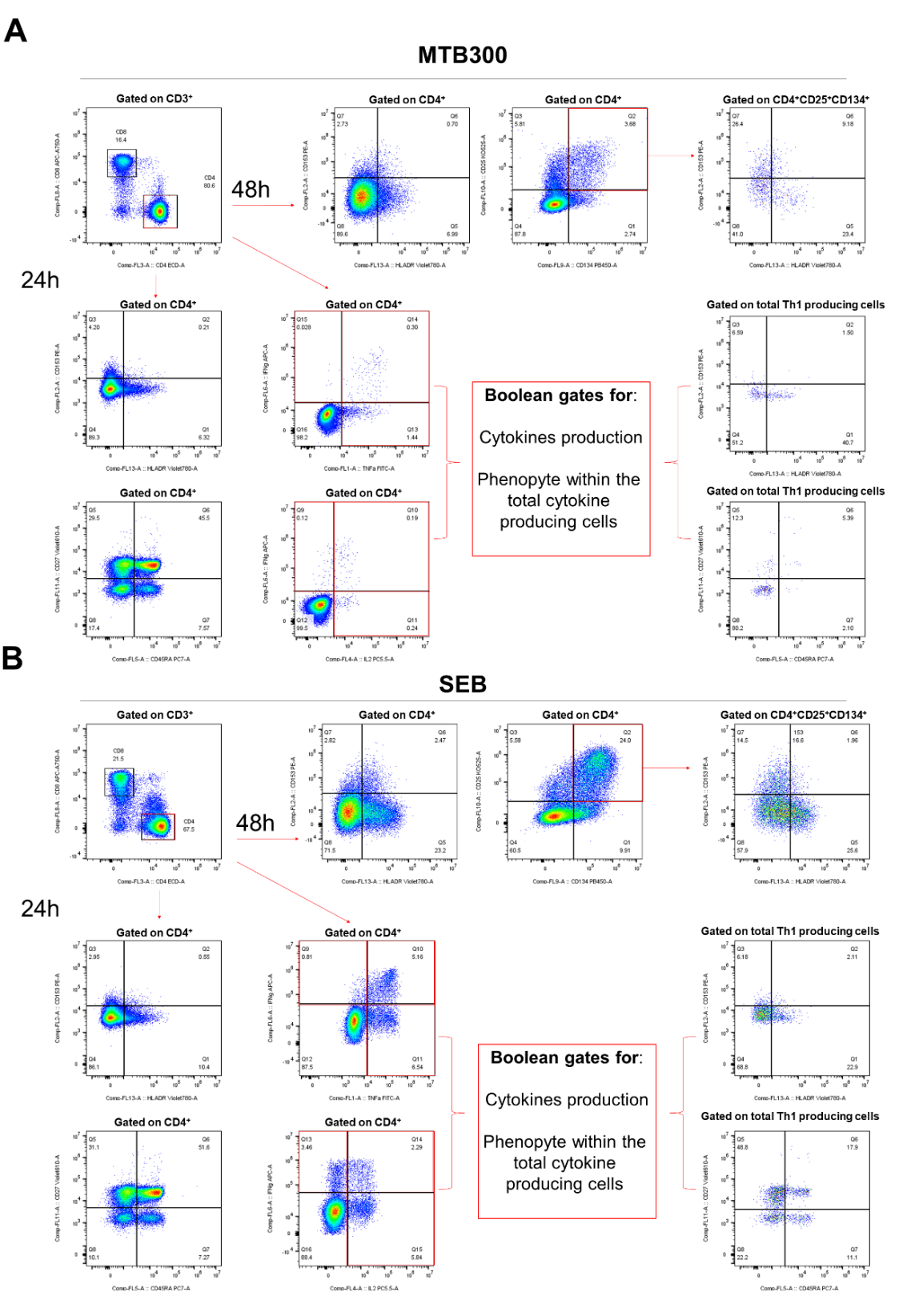


**Supplementary Figure S1**. Flow cytometry gating strategy for CD4 T-cell Th1 and CD25^+^CD134^+^ specific responses. PBMCs were stimulated with MTB300 (**A**) and SEB (**B**) as positive control and then stained as described in the methods section. The gating strategy shown is representative of a TBI-IMID subject.

**Supplementary Figure S2**


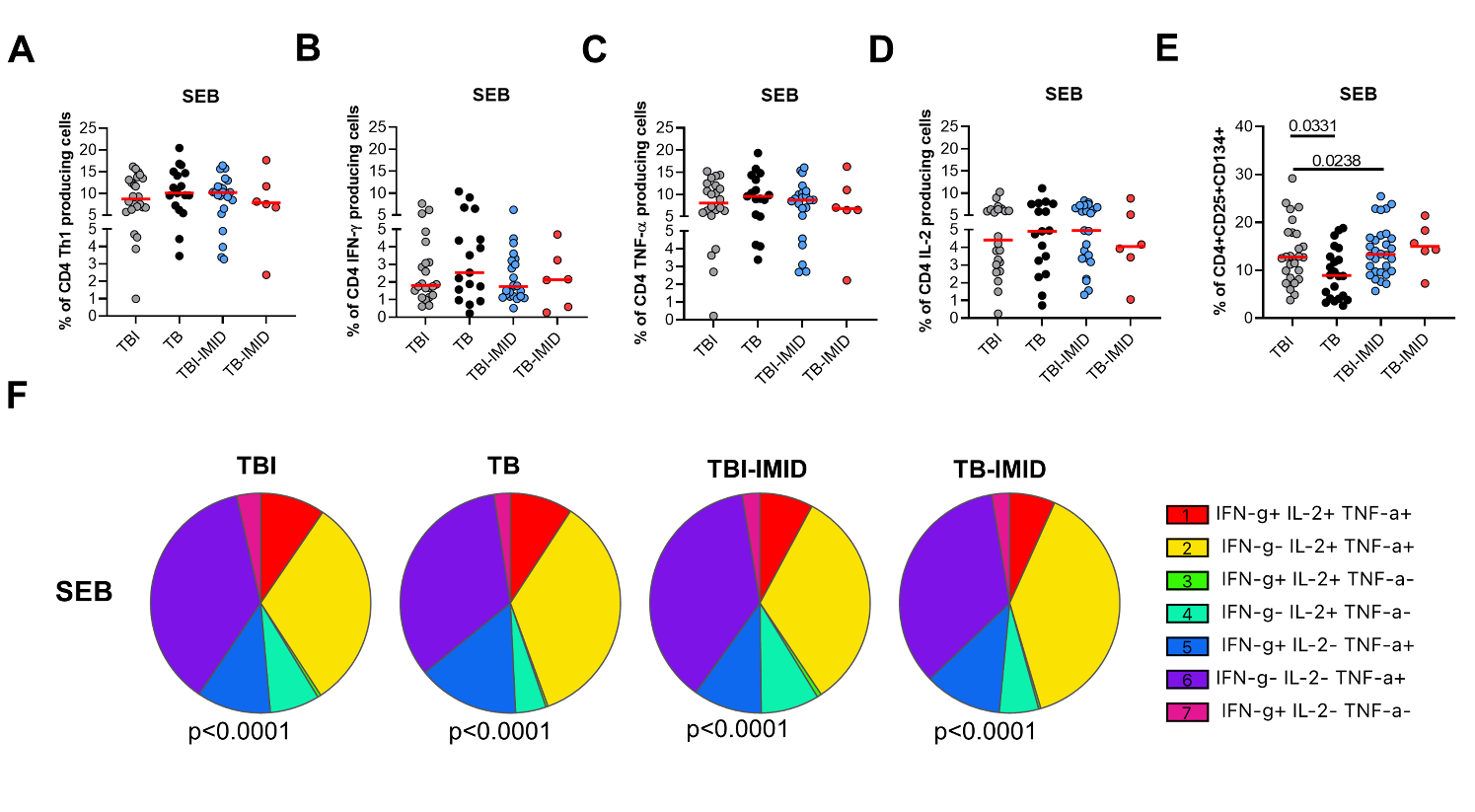


**Supplementary Figure S2. Evaluation of CD4 T-cell response to SEB in TB and TBI subjects with and without IMID.** PBMCs were stimulated with SEB for 24 or 48 hours and immune response was evaluated by flow cytometry. All the analyses were performed only among the responders. **A**) SEB response evaluated as total CD4 Th1 cytokine-producing cells after 24 h of stimulation. **B)** SEB response evaluated as total IFN-γ^+^ CD4^+^ T cells after 24 h of stimulation. **C)** SEB response evaluated as TNF-α^+^ CD4^+^ T cells after 24 h of stimulation. **D**) SEB response evaluated as IL-2^+^ CD4+ T cells after 24 h of stimulation. **E**) SEB response evaluated as CD25^+^ CD134^+^ CD4^+^ T cells after 48h of stimulation. **A-E)**. Horizontal red lines indicate the median and each dot represents a single subject. Statistical analysis was performed using the Mann-Whitney test. **F)** Pie charts representing the proportion of different cytokine-producing CD4^+^ T-cell subsets; boolean gate combination and Wilcoxon matched-pairs signed rank test were applied. Footnotes: TB: tuberculosis; TBI: tuberculosis infection; IMID: immune-mediated inflammatory disease; SEB: staphylococcal enterotoxin b; IFN-γ: interferon-gamma; IL-2: interleukine 2; TNF-α: tumor necrosis factor alpha.

**Supplementary Figure S3**

**
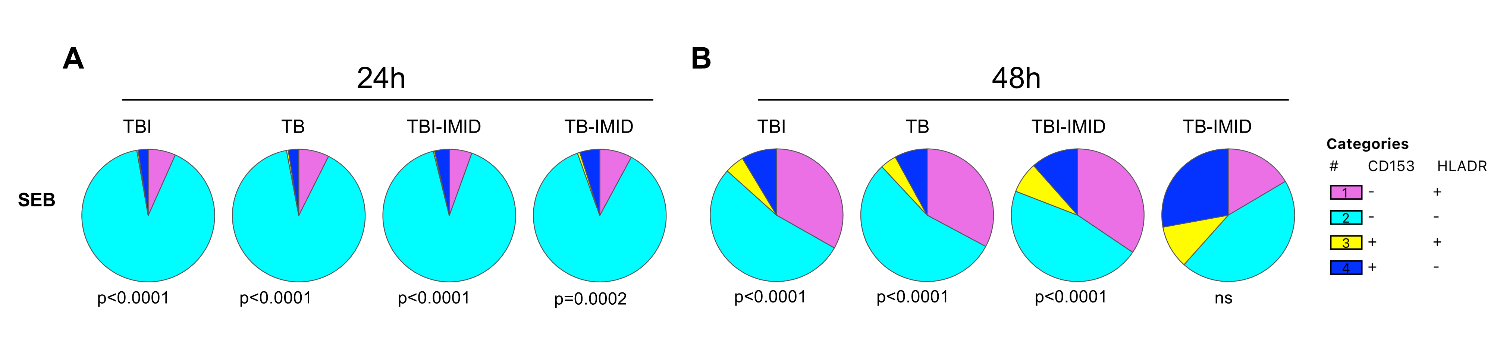
**

**Supplementary Figure S3. CD153 and HLA-DR expression in response to SEB stimulation in TB and TBI subjects with and without IMID after 24 and 48 hours of stimulation.** PBMCs were stimulated with SEB for 24 or 48 hours and the immune response was evaluated by flow cytometry. **A)** The immune response was evaluated after 24 hours of stimulation and defined as total CD4 Th1 cytokine-producing cells; the expression of CD153 and HLA-DR was evaluated only among the responders. **B)** The immune response was evaluated after 48 hours of stimulation and defined as CD25^+^ CD134^+^ CD4^+^ T cells; the expression of CD153 and HLA-DR was evaluated only among the responders. Pie charts representing the proportion of different CD4^+^ T-cell subsets. Boolean gate combination and Wilcoxon matched pairs signed rank test were applied. Footnotes: TB: tuberculosis; TBI: tuberculosis infection; IMID: immune-mediated inflammatory disease; SEB: staphylococcal enterotoxin b.

**Supplementary Figure S4**

**
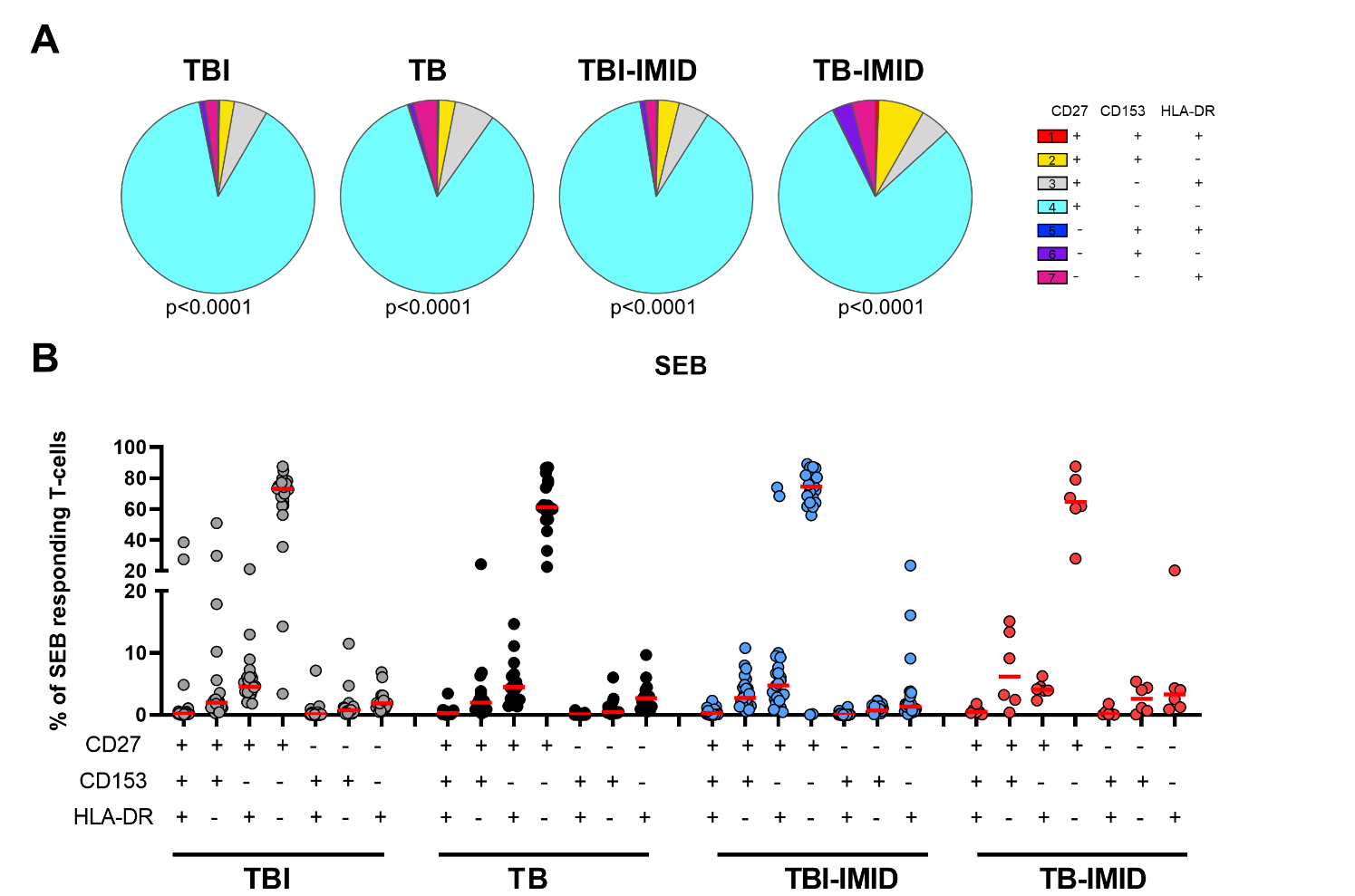
**

**Supplementary Figure S4. Activation profile of CD4^+^ T cells in response to SEB in TB and TBI subjects with and without IMID.** PBMCs were stimulated with SEB for 24 hours and the immune response was evaluated by flow cytometry. SEB response was defined as total CD4 Th1 cytokine-producing cells and the activation profile was evaluated only among the responders. **A)** Pie charts representing the proportion of CD27^+/-^ CD153^+/-^HLADR^+/-^ CD4 T-cell subsets; Wilcoxon matched-pairs signed rank test was applied **B**) Frequency of CD27^+/-^ CD153^+/-^HLADR^+/-^ CD4 T-cell subsets. Horizontal red lines indicate the median and each dot represents a single subject. Statistical analysis was performed using the Mann-Whitney test. Footnotes: TB: tuberculosis; TBI: tuberculosis infection; IMID: immune-mediated inflammatory disease

**Supplementary Figure S5**


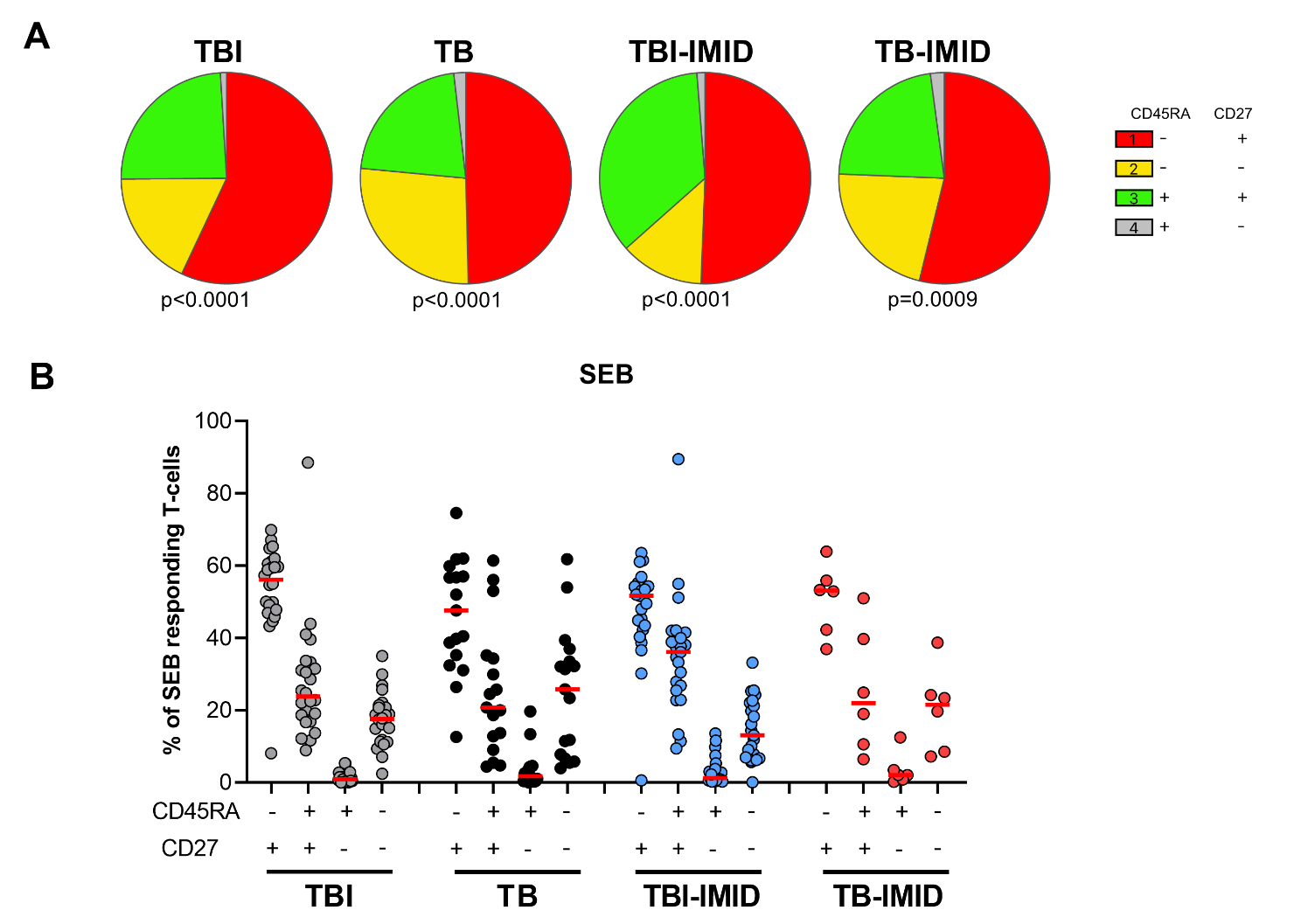


**Supplementary Figure S5. Memory profile of CD4 T-cell in response to SEB in TB and TBI subjects with and without IMID.** PBMCs were stimulated with SEB for 24 hours and the immune response evaluated by flow cytometry. SEB response was defined as total CD4 Th1 cytokine producing cells and the activation profile was evaluated only among the responders. **A)** Pie charts representing the proportion of CD27^+/-^ CD45RA^+/-^ CD4 T cell subsets; Wilcoxon matched-pairs signed rank test were applied **B**) Frequency of CD27^+/-^ CD45RA^+/-^ CD4 T cell subsets. Horizontal red lines indicate the median and each dot represents a single subject. Statistical analysis was performed using the Mann Whitney test. Footnotes: TB: tuberculosis; TBI: tuberculosis infection; IMID: immune mediated inflammatory disease

**Supplementary Figure S6**

**
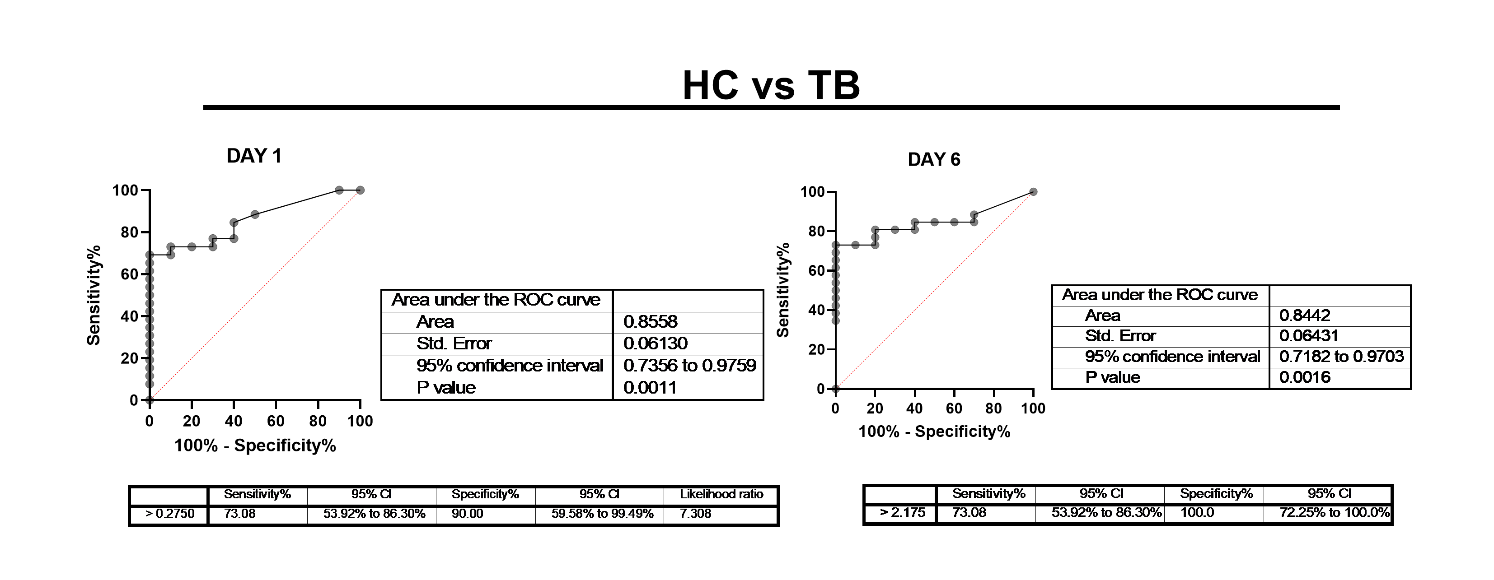
**

**Supplementary Figure S6. ROC analysis of IFN-γ production in response to MTB300 in healthy control versus TB patients.** PBMCs were stimulated with Mtb-specific antigens (MTB300) for 6 days in presence of IL-2. IFN-γ was evaluated by ELISA on day 1 and day 6 on supernatants in TBI-IMID QFT-Plus negative and QFT-Plus positive. A ROC analysis was applied to identify the cut-off of positivity for MTB300 stimulation for day 1 stimulation **(A)** and day 6 stimulation **(B).** AUC sensitivity and sensibility were calculated for the identification of TB patients. TB: tuberculosis; HC: healthy control; AUC: area under the curve; ROC receiver operating characteristic curve.
